# Inhibitory Role of an *Aeromonas hydrophila* TIR Domain Effector in Antibacterial Immunity by Targeting TLR Signaling Complexes in Zebrafish

**DOI:** 10.3389/fmicb.2021.694081

**Published:** 2021-07-08

**Authors:** Huai-ping Tang, Chen Huang, Chong-bin Hu, Hao Li, Tong Shao, Jian-fei Ji, Jun Bai, Dong-dong Fan, Ai-fu Lin, Li-xin Xiang, Jian-zhong Shao

**Affiliations:** ^1^College of Life Sciences, Key Laboratory for Cell and Gene Engineering of Zhejiang Province, Zhejiang University, Hangzhou, China; ^2^Laboratory for Marine Biology and Biotechnology, Qingdao National Laboratory for Marine Science and Technology, Qingdao, China

**Keywords:** TIR domain effector, *A. hydrophila*, TLR signaling pathways, CD80/86, antibacterial immunity

## Abstract

The Toll/interleukin-1 receptor (TIR) domain is a structural unit responsible for the assembly of signal protein complexes in Toll-like receptor (TLR) and interleukin-1 receptor signaling pathways. TIR domain homologs are found in a considerable number of bacteria and enhance bacterial infection and survival in host organisms. However, whether TIR domain homologs exist in *Aeromonas hydrophila*, a ubiquitous waterborne bacterium in aquatic environments, remains poorly understood. In this study, a TIR domain protein (TcpAh) was identified from *A*. *hydrophila* JBN2301. TIR domain of TcpAh is highly homologous to the counterpart domains in TLRs and myeloid differentiation factor 88 (MyD88). The zebrafish infected with mutant *A*. *hydrophila* with *tcpAh* deletion had a remarkably lower mortality than those infected with the wild-type strain. This result suggests that TcpAh is a crucial virulence factor for *A*. *hydrophila* infection. TcpAh exhibited a strong ability to associate with MyD88, tumor necrosis factor receptor-associated factor 3 (TRAF3) and TRAF-associated NF-κB activator-binding kinase 1 (TBK1) in TIR–TIR, TIR–Death domain (DD), and other alternative interactions. This finding suggests that TcpAh extensively interferes with MyD88 and TIR domain-containing adapter inducing interferon (IFN)-β (TRIF) signaling pathways downstream of TLRs. Consequently, CD80/86 expression was suppressed by TcpAh via attenuating TLR-stimulated NF-κB activation, which ultimately led to the impairment of the major costimulatory signal essential for the initiation of adaptive humoral immunity against *A*. *hydrophila* infection. We believe that this study is the first to show a previously unrecognized mechanism underlying *A*. *hydrophila* evades from host antibacterial defense by intervening CD80/86 signal, which bridges innate and adaptive immunity. The mechanism will benefit the development of therapeutic interventions for *A*. *hydrophila* infection and septicemia by targeting TcpAh homologs.

## Introduction

Toll-like receptors (TLRs) and interleukin-1 receptors (IL-1Rs) play crucial roles in an array of host immune responses (Tobias et al., 2000; Dunne and O’Neill, 2003). Signal transduction through these receptors leads to the activation of various transcription factors, such as NF-κB and activator protein 1 (AP-1) (Dunne and O’Neill, 2003). These two families of receptors share a conserved intracellular region with approximately 200 amino acids, known as the Toll/interleukin-1 receptor (TIR) domain (Slack et al., 2000; Akira and Takeda, 2004). This domain is also shared by the downstream signaling proteins, such as myeloid differentiation factor 88 (MyD88), TIR domain-containing adaptor protein (TIRAP), TIR domain-containing adapter inducing interferon (IFN)-β (TRIF) and TRIF-related adaptor molecule (TRAM), as a unit responsible for the signal-dependent assembly of protein complexes that enable the amplification and spatial propagation of a signal (O’Neill and Bowie, 2007). Site-directed mutagenesis and deletion analysis showed that the TIR domain is essential for TLR and IL-1R activities (Poltorak et al., 1998; Underhill et al., 1999). The TIR domain consists of three functional boxes of conserved residues set in a core sequence that ranges from 135 to 160 amino acids (Xu et al., 2000). Box 1 and 2 motifs participate in the association of proteins involved in signaling, whereas box 3 is involved in directing the localization of receptors (Slack et al., 2000; Xu et al., 2000). Two interfaces are responsible for mediating TIR domain interactions, which include receptor/adaptor oligomerization and the association between receptors and adaptors (Dunne et al., 2003). Crystal structure analysis showed that the TIR domains from human TLR1/2 contain a central five-stranded parallel β-sheet that is surrounded by five helices on both sides (Xu et al., 2000; Dunne et al., 2003). Conserved residues are located in the hydrophobic core and large insertions or deletions are present in several loop regions of different TIR domains. The BB loop, contains three highly conserved residues, protrudes from a large conserved surface patch, which is believed to mediate heterodimeric interactions with TIR domain-containing adaptor proteins (Xu et al., 2000; Dunne et al., 2003; Jin and Lee, 2008).

Among the numerous TIR domain-containing adaptors, MyD88 is the common adaptor recruited by all TLRs except TLR3 (Janssens and Beyaert, 2002). TIRAP is a unique adapter in TLR2 and TLR4 signaling and is associated with MyD88 for NF-κB activation (Horng et al., 2002). TRIF and TRAM activate IFN regulatory factor (IRF)-3, IRF-7, and NF-κB-dependent signaling pathways (Yamamoto et al., 2002; Oshiumi et al., 2003). TRIF functions downstream TLR3 and TLR4 signaling pathways, whereas TRAM is restricted to the TLR4 pathway (Yamamoto et al., 2003a,b). Some negative regulators, including single immunoglobulin IL-1 receptor related protein (SIGIRR), MyD88s, interleukin-1 receptor-associated kinase (IRAK)-M, Triad3A, and sterile alpha and TIR motif-containing protein (SARM), which block MyD88- or TRIF-dependent signaling pathways, co-evolve in host organisms to avoid excess inflammatory reactions (Kobayashi et al., 2002; Burns et al., 2003; O’Neill, 2003; Wald et al., 2003; Akira and Takeda, 2004; Carty et al., 2006; Fearns et al., 2006). The TIR domain in SIGIRR resembles MyD88 but lacks two amino acids needed for downstream signaling (O’Neill, 2003). In addition, the TIR–TIR interaction between SIGIRR and TLR4 prevents the recruitment of IRAK and tumor necrosis factor receptor-associated factor (TRAF) 6 to MyD88 (Wald et al., 2003). MyD88s is an alternatively spliced variant of MyD88 that lacks the intermediary domain; thus, it is unable to bind to IRAK4 and promote IRAK1 phosphorylation (Burns et al., 2003). IRAK-M prevents the dissociation of IRAK1–IRAK4 complex from MyD88 to prevent the formation of IRAK1–TRAF6 complex (Kobayashi et al., 2002). Triad3A interacts with the TIR domains of TLRs, TRIF, TIRAP and receptor-interacting protein 1 (RIP1); SARM blocks gene induction downstream of TRIF (Carty et al., 2006; Fearns et al., 2006). Interestingly, some of these negative regulatory strategies are imitated by bacterial TIR domain proteins to impair host TLR- and IL-1R-mediated signaling pathways and create a permissive environment facilitating bacterial infection and survival (Newman et al., 2006; Rosadini and Kagan, 2015). This observation implicates the complex evolutionary correlation between host and microbe.

Toll/interleukin-1 receptor domain proteins have been found in a considerable number of bacteria, such as *Salmonella enterica*, *Brucella melitensis*, *Escherichia coli* CFT073, *Yersinia pestis*, *Paracoccus denitrificans*, *Staphylococcus aureus* and *Pseudomonas aeruginosa* (Newman et al., 2006; Low et al., 2007; Cirl et al., 2008; Rana et al., 2011; Askarian et al., 2014; Imbert et al., 2017). These proteins include TlpA in *S*. *enterica*, TcpB in *B*. *melitensis*, TcpC in *E*. *coli* CFT073, YpTdp in *Y*. *pestis*, PdTLP in *P*. *denitrificans*, TirS in *S*. *aureus* and PumA in *P*. *aeruginosa*. These proteins enhance bacterial colonization and survival in host organisms. However, whether the TIR domain proteins exist in *Aeromonas hydrophila* remains poorly understood. *A*. *hydrophila* is one of the most ubiquitous waterborne bacteria in freshwater and brackish water environments. *A*. *hydrophila* shows wide host tropism, is frequently encountered in fish and other aquatic organisms, and is accountable for various infections, including severe aeromonad septicemia (Harikrishnan et al., 2003; Vivas et al., 2004). Therefore, understanding the associated mechanisms underlying *A*. *hydrophila* infection and pathogenesis has long been an attractive research topic waiting to be explored. In the present study, we identified a previously uncharacterized TIR domain protein (TcpAh) from *A*. *hydrophila* JBN2301 strain. TcpAh can strongly inhibit TLR signaling pathways by a wider association with MyD88, TRAF3 and TBK1 than previously known. TcpAh can also inhibit adaptive humoral immunity by downregulating CD80/86 costimulatory signal, which is essential for initiating CD4^+^ T cell activation and downstream B cell proliferation and antibody production (Greenfield et al., 1998; Pasare and Medzhitov, 2004; Crotty, 2011). We believe that this study is the first to report the existence of TIR domain protein-mediated mechanisms underlying *A*. *hydrophila* infection and adds a new member to the bacterial TIR domain protein family.

## Materials and Methods

### Bacterial Strain and Culture

*Aeromonas hydrophila* JBN2301 strain (accession number CP013178.1) was kindly provided by Prof. Yang of Wuhan Polytechnic University, Hubei province of China. This *A*. *hydrophila* strain was routinely grown in Tryptic Soy Agar (TSA) plates or Tryptic Soy Broth (TSB) at 28°C. Antibiotics were added to the bacterial cultures, when appropriate, at the following concentrations: 100 μg/ml tetracycline (Tc), 100 μg/ml ampicillin (Amp) and 30 μg/ml Chloramphenicol (Cm). *Escherichia coli* was grown in Luria-Bertani (LB) medium and antibiotics were added when necessary at the following concentrations: 50 μg/ml kanamycin (Km) and 100 ug/ml ampicillin. To measure the growth curve, *A*. *hydrophila* strain cultures were diluted 1:500 in TSB and grown at 28°C for 12 h at 200 rpm after overnight cultivation. Samples were taken hourly and measured at OD_600_. The bacterial experiments were performed followed by standard biosecurity and institutional safety procedures.

### Experimental Fish and Embryo

Wild-type AB zebrafish (*Danio rerio*) of both sexes and with body weights of 0.5–1.0 g was raised in our laboratory in recirculating water at 26–28°C under standard conditions. The fish were fed with commercial pellets at a daily ration of 0.7% of their body weight and held for at least two weeks prior to use in experiments for the evaluation of their overall health. Only healthy fish, as determined by their normal appearance and level of activity, were used in the study. Zebrafish embryos were prepared according to previous protocols (Ma et al., 2018). The animal experiments were performed in accordance with legal regulations and approved by the Committee on Animal Care and Use and the Committee on the Ethic of Animal Experiments of Zhejiang University.

### Bioinformatics Analysis

Genome location of the *tcpAh* gene was retrieved from the Genome Data Viewer in the National Center for Biotechnology Information (NCBI) database. Primers for gene cloning were predicted by the Primer-BLAST program. Multiple alignment of TIR domains were performed using Jalview/ClustalW and secondary structures were predicted by Jalview/Jpred. The 3D structures of TIR domains were predicted using Phyre server and figures were generated by PyMol.

### Plasmid Constructions

The full coding sequence of *tcpAh* was inserted into pET28a (Invitrogen) between the *Eco*RI and *Xho*I sites to construct the prokaryotic expression vector pET28a-*tcpAh*. For the construction of plasmids pET28a-CP-*tcpAh*, the MTS sequence (GCAGCCGTTCTTCTCCCTGTTCTTCTTGCCGCACCC) (Rojas et al., 1998) were synthesized and ligated to the carboxyl-terminal of *tcpAh* and then inserted into the pET28a vector by *Eco*RI and *Xho*I sites. For the construction of eukaryotic expression plasmids, the sequence encoding the full length of *tcpAh* was subcloned into pRFP-C1 (constructed in this study) and pcDNA6-Myc/His (Sigma-Aldrich), to obtain pRFP-C1-*tcpAh* and pcDNA6-*tcpAh* vectors for expressing recombinant TcpAh proteins with RFP- and Myc-tag, respectively. The encoding sequences of zebrafish MyD88, MyD88-TIR, MyD88-DD and TRIF were subcloned into pcDNA3.1-EGFP/HA/His (Invitrogen). The encoding sequences of zebrafish TRIF, TRAF3, and TBK1 were subcloned into pEGFP-C1 or pCMV-tag2b-Flag (Invitrogen). For luciferase reporter assays, the *cd*80/86 promoter-luciferase reporter was constructed in our laboratory. The zebrafish IFNφ1 promoter-luciferase reporter (IFNφ1-Luc) was previously constructed in our laboratory and the zebrafish IFNφ2 promoter-luciferase reporter (IFNφ2-Luc) was kind gift of Li laboratory (Lu et al., 2016; Ma et al., 2018). The human NF-κB, IRF3, and IFN-β luciferase reporters and pRL-TK renilla luciferase reporter vectors were purchased from Clontech and Promega, respectively (Ma et al., 2018). The primers used for construction are listed in Supplementary Table 2. All constructs were sequenced to verify the correct sequences and orientations. Plasmids for transfection and microinjection were prepared free of endotoxin using an EZNA plasmid mini kit (Omega Bio-Tek).

### Preparation of Recombinant Proteins

For eukaryotic expression of indicated proteins, the plasmid DNAs were transfected into HEK293T cells. For prokaryotic expression of soluble TcpAh or CP-TcpAh recombinant protein, the pET28a-*tcpAh* or pET28a-CP-*tcpAh* vectors were transformed into the BL21 (DE3) *E*. *coli* strain (Novagen). The freshly transformed cells were grown in LB Broth (supplemented with 50 μg/mL of kanamycin) until an OD_600_ of 0.8 was reached at 37°C. IPTG (0.2 mM) was added to induce protein expression for 16 h at 20°C. After ultrasonication, the supernatants were collected for purification. The recombinant TcpAh-His or CP-TcpAh-His proteins were purified by nickel–nitrilotriacetic acid agarose affinity chromatography (Qiagen), following the manufacturer’s manual, and then detected by 12% SDS–PAGE.

### Preparation of Polyclonal Antibody

Four-week-old male ICR mice (∼15 g) were immunized with the recombinant TcpAh protein (20 μg) each time in CFA (Sigma- Aldrich) initially and then in IFA (Sigma-Aldrich) for four times thereafter at biweekly intervals, as previously described (Shao et al., 2018). Seven days after the final immunization, serum samples were collected. Antibody against TcpAh were affinity purified by using Protein A agarose columns (Thermo Fisher Scientific), and their titers were examined by Enzyme-Linked Immunosorbent Assay (ELISA). The validity and specificity of the antibody were determined by Western blot analysis. The Abs against zebrafish MHC class II (MHC-II), mIgM, CD80/86, including rabbit anti-MHC-II, mouse anti-mIgM, rabbit anti-CD80/86, were produced in our previous studies (Shao et al., 2018).

### Construction of *A. hydrophila* Δ*tcpAh* Mutant

To generate Δ*tcpAh* mutant, the upstream and downstream flanking sequences to *tcpAh* were amplified by PCR using two pairs of primers P1/P2 and P3/P4 as shown in Supplementary Table 2. In addition, a Tc^*r*^ gene cassette was amplified from the plasmid pBBR1MSC-3 (MiaoLing Plasmid Sharing Platform) using primers P5/P6 (Supplementary Table 2). The resulting 1148-, 1184- and 1267-bp DNA fragments were ligated together through overlap PCR and subcloned into pRE112 (MiaoLing Plasmid Sharing Platform) suicide vector at *Kpn*I restriction enzyme site using a Clone Express^®^ II One Step Cloning Kit (Vazyme) to obtain a pRE112-Δ*tcpAh*Tc^*r*^ plasmid. Then, the pRE112-Δ*tcpAh*Tc^*r*^ was transformed into *E*. *coli* SM10 λpir (MiaoLing Plasmid Sharing Platform) for mobilization into wild-type *A*. *hydrophila* via conjugation. The resultant Δ*tcpAh* mutant strain was selected by tetracycline and sucrose resistance after allelic exchange between the chromosomal gene and the mutagenized plasmid copy by twice crossover event, and was verified by PCR and DNA sequencing and subjected to further analysis by Western blot analysis (Xie et al., 2018).

### Challenge Assay

Wild-type *A*. *hydrophila* JBN2301 and Δ*tcpAh* mutant were collected from the logarithmic growth phase, and zebrafish were inoculated i.p. with the wild-type and mutant *A*. *hydrophila* at concentration of 5 × 10^6^ CFU/fish (for 96 h challenge assay) or 2 × 10^4^ CFU/fish (for 21 days challenge assay). In these cases, mock PBS was administered as negative control. Infection and mortality in each group was monitored for 96 h at one interval 12 h, or during the 21 days period at one interval 2 days. Kaplan–Meier survival curve was obtained using GraphPad Prism software version 8.0. Statistical differences between wild-type versus Δ*tcpAh A*. *hydrophila* infected groups were analyzed using log-rank test. Bacterial load was detected in gill, spleen, and kidney tissues of zebrafish in each group. For this, the tissues were collected and washed with sterilized PBS, homogenized, and centrifuged at 5,000 *g* at 4°C for 10 min. Bacterial load was calculated and expressed into colony forming unit (CFU) by counting the colonies appearing on the TSA plates as described (Saraceni et al., 2016).

### *In vivo* Luciferase Reporter Assays

*In vivo* luciferase reporter assays were performed to examine MyD88-dependent TLR2- or TLR9-signaling activation and TRIF-dependent TLR3-signaling activation in zebrafish embryos through overexpression objective genes. For this, the one-cell-stage embryos were microinjected (2 nL) with 100 pg NF-κB-Luc or zebrafish IFNφ1/φ2-Luc reporter vectors, and 10 pg renilla luciferase reporter vectors with increasing amounts (0, 50, 100 pg) of pcDNA6-*tcpAh* vector. Empty control vector was added so that a total of 360 pg vector DNA was microinjected into each embryo. At 12 h post microinjection (hpm), embryos were stimulated with Pam3CSK4 (200 pg/embry; Invivogen), CpG-ODN (400 pg/embryo; Generay Biotechnology Company), TNF-α (10 pg/embryo; eBiosience), and poly (I:C) (200 pg/embryo; Invivogen) for 12 h, respectively. Plasmid DNAs were mixed in a microinjection buffer (0.5% phenol red, 240 mM KCl, and 40 mM HEPES, pH 7.4). Luciferase activity in total embryo lysates was detected with a Dual Luciferase Reporter Assay (Promega) at 24 hpm.

### *In vitro* Luciferase Reporter Assays

*In vitro* luciferase reporter assays were performed to examine MyD88-mediated activation of NF-κB or *cd80/86* promoter and TRIF-mediated activation of IRF3 or IFN-β signaling in HEK293T cells. HEK293T cells were seeded in 12-well plates at a density of 5 × 10^5^ cells/ml for 24 h and co-transfected with various indicated plasmids, luciferase reporter plasmid, and control reporter plasmid. pRL-TK vector was used as an internal control to normalize the expression level of the transfected plasmid. At 24 h post transfection, cells were stimulated with indicated stimulants for 12 h. Then cells were washed with PBS, lysed with Passive Lysis Buffer (Promega), and assayed for luciferase activities in a luminometer by the Dual-Luciferase Reporter Assay System (Promega). The luciferase reading of each sample was first normalized against that in the pRL-TK level, and the relative light unit was presented as the ratio of firefly luciferase to renilla luciferase. The results were obtained from three independent experiments (Ji et al., 2019).

### Co-immunoprecipitation (Co-IP) and Western Blot Analysis

HEK293T cells were cultured in dishes with a diameter of 100 mm at 37°C in 5% CO_2_ overnight. Cells were transiently transfected with 24 μL of PEI transfection reagent (Promega) containing a total of 6 μg plasmid DNAs, including pcDNA6-*tcpAh* plus pcDNA3.1-MyD88 or pcDNA3.1-MyD88-TIR or pcDNA3.1-MyD88-DD (at a ratio of 1:1); pcDNA6-*tcpAh* plus pCMV-TRIF or pCMV-TRAF3 or pCMV-TBK1 (at a ratio of 1:1) in different combinations, with the empty vector as control. After 48 h, the cells were washed with PBS and lysed for 30 min at 4°C in an ice-cold buffer containing 20 mM Tris–HCl (pH 7.5), 150 mM NaCl, 1 mM EDTA, 1% Triton X-100, and the cocktail protease inhibitor (Roche). Cell lysates were centrifuged at 4°C, 13,000 *g* for 10 min and the supernatants were incubated with mouse anti-Myc or anti-Flag tag mAb (Abmart; M20002M, M20008L) at 4°C overnight, followed by incubation with 50 μL protein A-agarose beads (Roche) for 3 h. Then, the beads were washed four times with lysis buffer. The precipitants were denatured in loading buffer for analysis by Western blot using 12% SDS–PAGE and transferred onto 0.22 μm polyvinylidene difluoride membranes (Bio-Rad). The blots were probed with mouse/rabbit anti-Myc (Abmart; M20002L, Sangon biotech; D110006-0200) or GFP (Abmart; M20004L, HuaBio; ET1607-31) or Flag (Abmart; M20008L, Sangon biotech; D110005-0200) tag Abs at 1:5,000 and HRP-conjugated goat anti-mouse/rabbit IgG (Abmart; M21001S, M21002S) at 1:8,000, and then incubated with ECL reagents (Millipore) according to the manufacturer’s instructions. The emitted light was detected using a cooled CCD camera (LAS–1000; Fuji film) (Ma et al., 2018).

### Fluorescence Localization Imaging

HEK293T cells were seeded into multiwell plates (Corning) and cultured in DMEM (HyClone Laboratories) supplemented with 10% FBS, 100 U/ml penicillin, and 100 μg/ml streptomycin at 37°C in 5% CO_2_ to allow growth into 70–90% confluence. These cells were co-transfected with RFP tagged TcpAh plasmid DNA (500 ng) and a series of GFP tagged objective plasmid DNA (500 ng) using PEI in accordance with the manufacturer’s instructions (Wan et al., 2016). At 24 h post transfection, the cells were washed with PBS and fixed in 4% paraformaldehyde for 10 min and stained with 100 ng/ml DAPI (Sigma) at room temperature for 10 min. For localization imaging in stimulated HEK293T cells, the cells were co-transfected with RFP tagged TcpAh plasmid DNA (500 ng) and a series of GFP tagged objective plasmid DNA (500 ng) together with TLR2 or TLR3 plasmids (50 ng) using PEI in accordance with the manufacturer’s instructions. At 24 h post transfection, cells were stimulated with Pam3CSK4 or Poly(I:C) for 12 h, and then harvested as above described. Fluorescence images were obtained using a laser scanning confocal microscope (Zeiss LSM 710).

### Flow Cytometric (FCM) Analysis

Cells under examination were blocked with 1% goat serum for 1 h at 4°C and then incubated with the defined primary Abs for 1 h at 4°C. Non-specific rabbit or mouse IgG was served as the negative control. After washing twice with D-Hank’s buffer, the cells were incubated with secondary Abs (PE conjugated goat anti-mouse or FITC conjugated goat anti-rabbit) for 1 h at 4°C, and the fluorescence signals were determined using the flow cytometer (BD FACSCalibur). At least 10,000 cells were collected from the myelomonocyte or lymphocyte gate for analysis. Cell Quest software (BD Biosciences) and ModFit LT software were used for FCM analysis and T cell proliferation assays, respectively (Wan et al., 2016; Shao et al., 2018).

### *In vitro* Assay for TcpAh on Lymphocyte Proliferation and Activation

Zebrafish were i.p. injected with sterile PBS, wild-type *A*. *hydrophila* (2 × 10^4^ CFU/fish), Δ*tcpAh* mutant, or Δ*tcpAh* mutant supplemented with CP-TcpAh recombinant protein for 5 days before sacrificed. Leukocytes were sorted from spleen, head kidney, and peripheral blood through Ficoll-Hypaque density-gradient centrifugation as described (Wan et al., 2016). Leukocytes obtained from fish with infection of wild-type *A*. *hydrophila*, Δ*tcpAh* mutant, or Δ*tcpAh* mutant plus CP-TcpAh were subjected to MHC-II^+^ antigen-presenting cells (APCs) isolation. For this, the cells were blocked with 1% goat serum for 1 h at 4°C and incubated with rabbit anti-MHC-II Ab for 2 h at 4°C. After the incubation, the cells were gently washed thrice with D-Hank’s buffer, incubated with anti-rabbit IgG magnetic beads (Thermo Scientific) for 15 min at 4°C, and then applied to a magnetic separator to separate the MHC-II^+^ cells. The MHC-II^+^ cells were cultured in L-15 medium containing 10% FBS (Gibco), 100 U/mL penicillin, and 100 μg/mL streptomycin at 28°C overnight to detach the magnetic beads. In parallel, leukocytes from fish with stimulation of inactivated *A*. *hydrophila* were stained with 10 μM CFSE (Beyotime), and terminated by adding 10% FBS. Then, the MHC-II^+^ APCs were cocultured with the CFSE-labeled leukocytes for 72 h. The proliferation and activation of lymphocytes were examined via FCM, and the expression of CD154 and LCK was detected using real-time PCR (Shao et al., 2018).

### Real-Time PCR for Gene Expression Analysis

Total RNAs from leukocytes were isolated using TRIzol reagent (Invitrogen) and transcribed into first-strand cDNA with oligo(dT)18 (Takara Bio). Real-time PCR was performed using a Master cycler ep real plex instrument (Eppendorf) with a SYBR Premix Ex Taq kit (Takara Bio), following the manufacturer’s instructions. Briefly, the reaction mixtures in a total volume of 10 μl were incubated for 2 min at 95°C, followed by 40 cycles of 15 s at 95°C, 15 s at 60°C, and 20 s at 68°C. The relative gene expression of *cd80/86* or other genes was calculated using the 2^–Δ^ ^cycle threshold^ and 2^–Δ^ ^Δ^ ^cycle threshold^ methods with β-actin for normalization. In all cases, each PCR trial was performed with triplicate samples and repeated at least three times (Shao et al., 2018). The forward and reverse primers used were shown as in Supplementary Table 2 in Supplementary Material.

### Effect of TcpAh on B Cell Activation and IgM Production

For the B cell activation assay, zebrafish were i.p. injected with *A*. *hydrophila*, Δ*tcpAh* mutant or Δ*tcpAh* mutant supplemented with CP-TcpAh recombinant protein. After 5-day stimulation with indicated stains, leukocytes from the spleen, kidney, and peripheral blood were collected, and the proliferation and activation of B cells were examined as the increase of mIgM^+^ cells through FCM with mouse anti-mIgM Ab. For the IgM production assay, fish were i.p. immunized with *A. hydrophila*, Δ*tcpAh* mutant or Δ*tcpAh* mutant plus CP-TcpAh. Serum samples were collected at 14 days after the immunization, and the level of IgM against heat-inactivated *A*. *hydrophila* was detected by ELISA (Wan et al., 2016; Xu et al., 2016). Briefly, the heat-inactivated *A*. *hydrophila* was used to coat 96-well ELISA plate overnight at 4°C. Then, the coated plate was treated with 2% BSA for 1 h at 37°C and washed with PBST (PBS with 0.05% Tween-20). Thereafter, the plate was loaded with serially diluted serum samples at 37°C. After incubation for 2 h, the plate was washed thrice with PBST and incubated with mouse anti-IgM Ab for 1 h at 37°C. Afterward, the plate was washed, and the HRP-conjugated goat anti-mouse-IgG Ab was added. Color was developed using tetramethylbenzidine and stopped with 2 mol/L H_2_SO_4_, and then measured at 450 nm on a Synergy H1 Hybrid Reader (BioTek Instruments). Ab titer is defined as the highest dilution of serum at which the A_450_ ratio (A_450_ of post-immunization sera/A_450_ of pre-immunization sera) is greater than 2.1 (Xu et al., 2016).

### Statistical Analysis

Results were shown as mean ± SD from at least three independent experiments. Statistical evaluation of differences between means of experimental groups was performed using ANOVA and Student’s *t*-tests. Survival curve differences in the *A*. *hydrophila* challenge assay were assessed using log-rank test. Statistical significance was considered when *p* < 0.05 or *p* < 0.01 or *p* < 0.001. The sample number for each group of fish exceeded 10 and each group of zebrafish embryo exceeded 50.

## Results

### Molecular Identification of TcpAh From *A. hydrophila*

**FIGURE 1:**
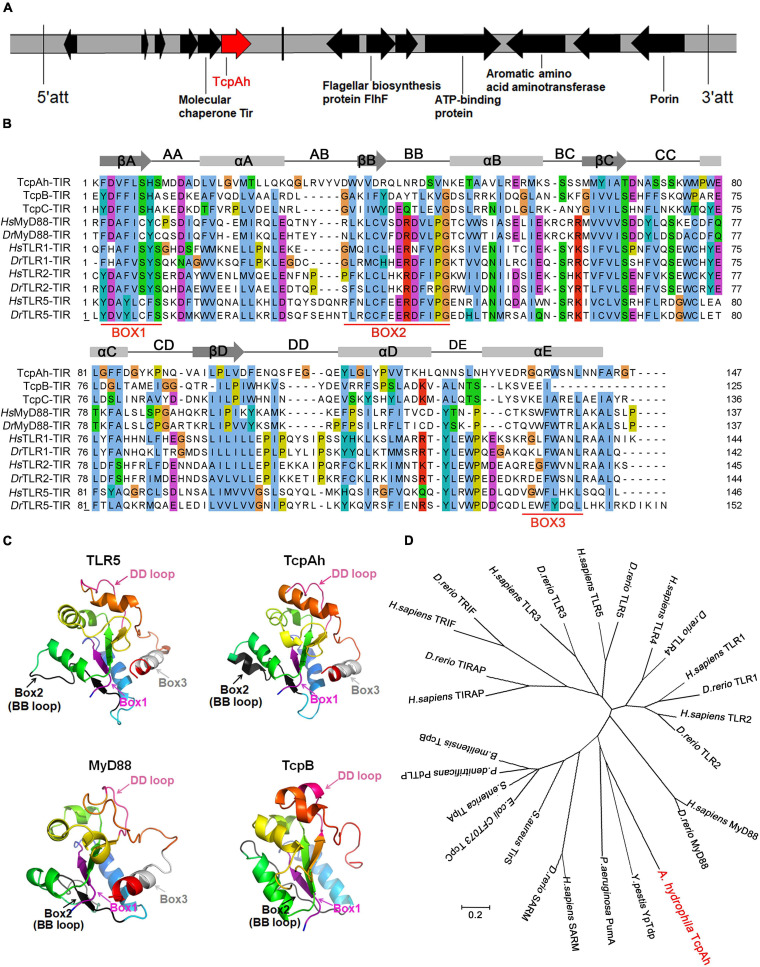
Bioinformatic characterization of TcpAh-encoding gene and protein in *A*. *hydrophila* JBN2301 strain. **(A)** Localization of TcpAh-encoding gene in JBN2301 genome. The encoding sequence of TcpAh is indicated in red arrow and att means attachment site. **(B)** Sequence alignment of TIR domains using Jalview/ClustalW and ClustalX programs. Conserved functional motifs and amino acid residues are shaded in different colors. Secondary structures were predicted by Jalview/Jpred program. Gray boxes, gray arrows, and black lines denote alpha helices, beta-sheet areas, and connecting loops, respectively. **(C)** Prediction of the 3D structures of TIR domains using Phyre server and PyMol program. The TcpAh TIR domain (38–184 aa) was modeled with 100% confidence by the single highest scoring template as the TIR domain of TLR5 (PDB c3j0aA). Box 1 (purple), box 2 and BB loop (black), and box 3 and DD loop (hot pink) are indicated within the TIR domains. **(D)** Phylogenetic trees of the amino acid sequences of TcpAh and its homologs in other species constructed by neighbor-joining method using MEGA 7.0. The tree was drawn to scale, and branch lengths are in the same units as those of the evolutionary distances used to infer the phylogenetic tree. The evolutionary distances were computed using the p-distance method. The GenBank accession numbers of the sequences are as follows: *H*. *sapiens* MyD88, AAC50954.1; *D*. *rerio* MyD88, AAQ91324.1; *H*. *sapiens* TLR1, NP_891549.1; *D. rerio* TLR1, AAI63271.1; *H*. *sapiens* TLR2, AAY85648.1; *D*. *rerio* TLR2, NP_997977.1; *H*. *sapiens* TLR3, NP_003256.1; *D*. *rerio* TLR3, AAI07956.1; *H*. *sapiens* TLR4, NP_003257.1; *D*. *rerio* TLR4, NP_001315534.1; *H*. *sapiens* TLR5, NP_003259.2; *D*. *rerio* TLR5, NP_001124067.2; *H*. *sapiens* TRIF, AAH09860.2; *D*. *rerio* TRIF, NP_001038224.1; *H*. *sapiens* TRIAP, NP_001034750.1; *D*. *rerio* TRIAP, XP_002667158.2; *H*. *sapiens* SARM, NP_055892.2; *D*. *rerio* SARM, NP_001124068.1; *Y*. *pestis* YpTdp, WP_198249346.1; *A*. *hydrophila* TcpAh, WP_043158960; *P*. *aeruginosa* PumA, WP_012075302.1; *S*. *aureus* TirS, WP_000114516.1; *E*. *coli CFT073* TcpC, WP_000282336.1; *S*. *enterica* TlpA, WP_079786625.1; *P*. *denitrificans* PdTLP, QAR27511.1; *B*. *melitensis* TcpB, EPZ76643.1.

A TIR domain protein (TcpAh)-encoding gene was retrieved from the genome database of *A*. *hydrophila* JBN2301 strain maintained by the National Center for Biotechnology Information (NCBI) through Basic Local Alignment Search Tool (BLAST) using a homologous sequence of *B*. *melitensis* TcpB as a query. This gene was named as *tcpAh* (accession number CP013178.1) because it originates from *A*. *hydrophila*. The *tcpAh* gene consists of 579 bp and located downstream a putative molecular chaperone Tir gene and upstream an unannotated sequence with 7.55 kb in length (Figure 1A). The *tcpAh* gene was predicted to encode TcpAh protein with 192 amino acids and molecular weight of 22 kDa. TcpAh contains a conserved TIR domain (38–184 aa) with 147 amino acids. The TIR domain of TcpAh is characterized by three functional boxes, namely, box 1 (F/Y) D–HS), box 2 (DW–VN) and box 3 (RG–NL), which also exist in other TIR domains from various bacterial Tcp proteins, as well as TLRs and adapters, such as MyD88 and TIRAP. Among the three boxes of TcpAh, box 1 shows the most similarity to those in other TIR domains. Box 1 contains several important residues, such as Phe/Tyr and Asp, which contribute to homodimer formation and are completely conserved in most TIR domains. By contrast, TcpAh lacks a well-defined box 2 region and some characteristic residues, such as the conserved Pro and Gly, which are critical for signaling in eukaryotic TIR domains. The box 3 region is also poorly conservative and lacks even the most highly conserved amino acid residues of the canonical TIR sequence (Figure 1B). Despite divergence in box 2 and box 3 regions, the secondary structure of TcpAh exhibits additional structure homologies in the BB and DD loops, which are functionally important for TIR domains (Figures 1B,C). In addition, tertiary architecture analysis showed that TcpAh shares considerable similarity with other TIR domains, particularly that of TLR5 (Figure 1C). The phylogenetic tree shows that TcpAh is clustered with other bacterial TIR domain proteins with high bootstrap probability, particularly with the YpTdp protein, which is a TIR domain protein in *Y*. *pestis* that inhibits NF-κB by interacting with MyD88 (Figure 1D). Notably, animal SARM is more closely related to bacterial TIR domain proteins than to other TIR domain proteins in animals, thus, an evolutionary correlation exists between SARM and bacterial TIR-domain proteins (Figure 1D). Importantly, TcpAh homologs were predicted in nine additional *A*. *hydrophila* strains with known genome sequences (Supplementary Table 1); and a number of highly conserved phage-signature sequences that encode site-specific integrases, coat and flagellar proteins were predicted in the regions flanking *tcpAh* homologous genes. These observations implied the possible phage-origin of the *tcpAh* homologs.

### TcpAh Is Required for *A. hydrophila* Infection

**FIGURE 2:**
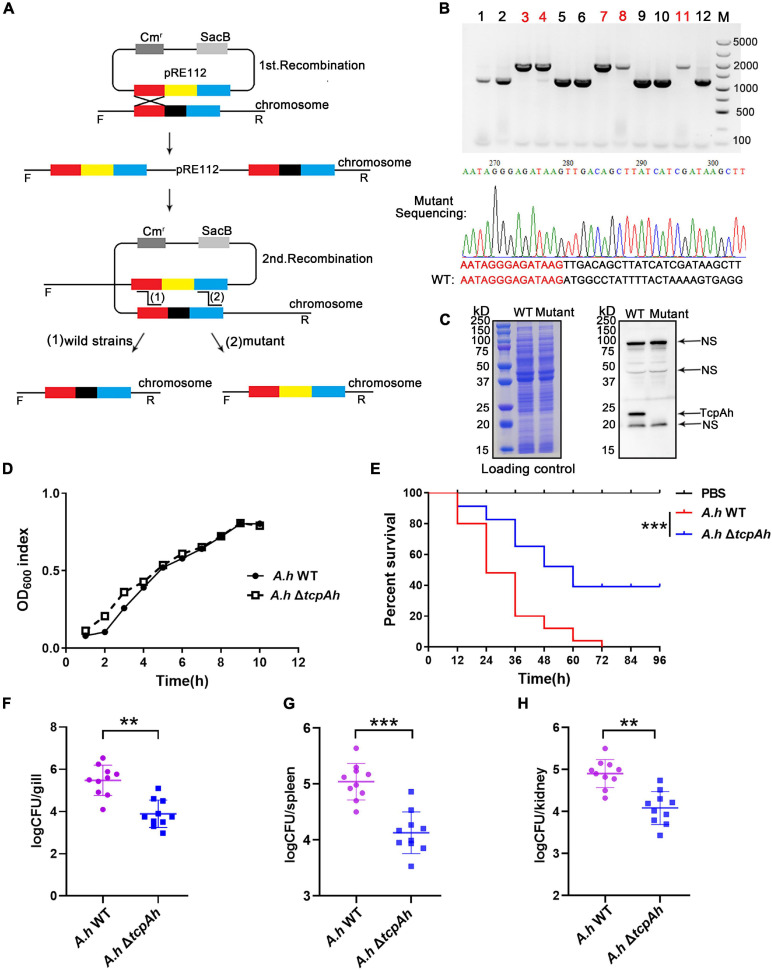
Examination of the requirement of TcpAh for *A. hydrophila* infection. **(A)** Strategy for the deletion of the *tcpAh* gene in *A*. *hydrophila* JBN2301 strain by homologous recombination. The black block indicates the *tcpAh* gene; red and blue blocks indicate the left and right homologous sequences of the *tcpAh* gene, respectively; and the yellow block indicates the Tc^*r*^ gene. **(B)** PCR and sequencing identification of the mutant strain with *tcpAh* gene deletion (Δ*tcpAh*). The lanes with red number indicate the mutant strain; partial sequencing result is showed. **(C)** Western blot analysis of TcpAh protein in wild-type and mutant *A*. *hydrophila* JBN2301 strains in liquid cultures and NS means non-specific band. The total proteins of wild-type and Δ*tcpAh A*. *hydrophila* JBN2301 strains were separated by SDS-PAGE and stained with Coomassie brilliant blue R250 as a loading control. The TcpAh protein was detected by using a polyclonal rabbit anti-TcpAh antibody with an expected molecular weight of 22 kDa. **(D)** Growth curve of wild-type and Δ*tcpAh A*. *hydrophila* JBN2301 strains. **(E)** Zebrafish survival curve. Zebrafish were infected with wild-type *A*. *hydrophila* JBN2301 and Δ*tcpAh* mutant. Statistical differences between wild-type versus Δ*tcpAh A*. *hydrophila* infected groups were analyzed by log-rank test. *n* = 25. ***p < 0.001. Group of fish injected with mock PBS was used as a negative control. **(F–H)** Examination of bacterial load in the **(F)** gill, **(G)** spleen, and **(H)** kidney tissues of zebrafish infected with wild-type and Δ*tcpAh* mutant *A*. *hydrophila* JBN2301 strains. Non-parametric two-tailed Mann–Whitney test was carried out with **(F)** ^∗∗^*p* < 0.01, (G)***p < 0.001, and **(H)** **p < 0.01.

A mutant *A*. *hydrophila* strain with *tcpAh* gene deletion (Δ*tcpAh*) was constructed by allelic replacement in wild-type (WT) JBN2301 strain to analyze the potential virulent activity of TcpAh in *A*. *hydrophila* infection. For this procedure, the up- and downstream flanking fragments of *tcpAh* in JBN2301 genomic DNA and a tetracycline resistance (Tc^*r*^) gene cassette in pBBR1MCS-3 DNA were amplified by polymerase chain reaction (PCR) using the primers shown in Supplementary Table 2. The fusion of the three fragments was amplified by overlap PCR and then ligated into suicide plasmid (pRE112) at the *Kpn*I sites. The resulting plasmid (pRE112-Δ*tcpAh*Tc^*r*^) was transformed into *E*. *coli* SM10 λpir for mobilization into JBN2301 via conjugation. The resultant Δ*tcpAh* mutant strain was selected by tetracycline and sucrose resistance after allelic exchange between the chromosomal gene and the mutagenized plasmid copy by twice crossover event (Figure 2A) and was verified by PCR and DNA sequencing (Figure 2B). The virulent activity of TcpAh was examined by a challenge assay in a well-established zebrafish infection model. Zebrafish were infected by intraperitoneal inoculation with wild-type *A*. *hydrophila* JBN2301 and Δ*tcpAh* mutant strains (5 × 10^6^ CFU/fish) and observed for 96 h. The results showed that the wild-type strain caused a remarkable lethality (52%) within 24 h and displayed 100% lethality 72 hours after infection. However, the Δ*tcpAh* mutant strain caused a remarkably lower lethality during the same time period after inoculation, and the final lethality remained at 56%, which is lesser by 44% than that of the wild-type strain (Figure 2E). Although indiscriminate clinical signs, such as depression, gill hyperemia, and accumulated ascites, were observed in the two infection groups, the delay of clinical-symptom appearance and the extended survival time in the mutant infection group suggested that the mutation of *tcpAh* substantially weakened the virulence of *A*. *hydrophila*. For further clarification, a bacterial load assay was performed in the different tissues of zebrafish infected with wild-type and Δ*tcpAh-*mutant *A*. *hydrophila*. The result showed that Δ*tcpAh* mutant infected zebrafish showed a decreased bacterial load in gill, spleen, and kidney tissues compared with that in wild-type strain infected fish (Figures 2F–H). The differences in survival rate and bacterial load between zebrafish infected with wild-type and Δ*tcpAh*-mutant *A*. *hydrophila* were not likely due to the defect in the growth of the mutant strain and abnormal protein expression in wild-type strain, because a similar growth rate was observed between the two strains and a highly expressed TcpAh protein level was detected in wild-type *A*. *hydrophila* (Figures 2D,C). The results indicate that TcpAh is a crucial virulence-related factor required for *A*. *hydrophila* infection.

### TcpAh Inhibits MyD88 Signaling Pathway

**FIGURE 3:**
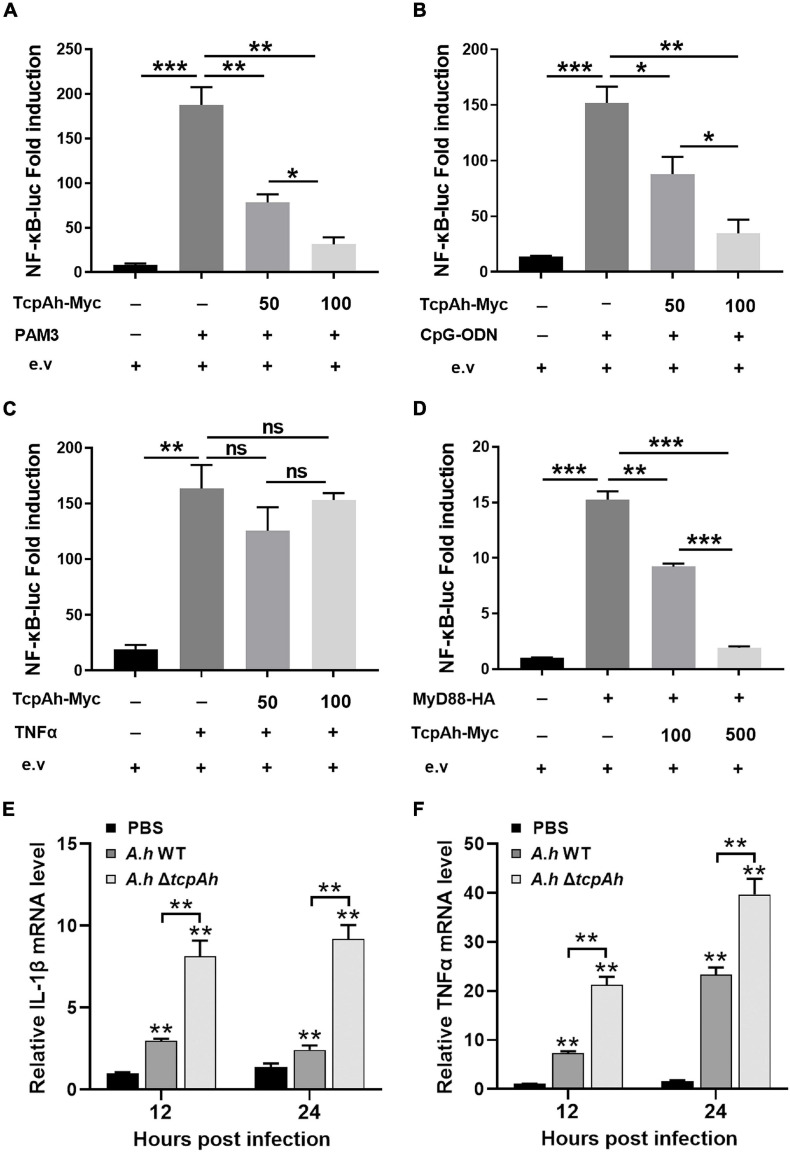
Examination of the inhibitory role of TcpAh in MyD88 signaling pathway. **(A–C)** Activation of the NF-κB-binding promoters detected in zebrafish embryos microinjected with NF-κB luciferase reporter (NF-κB-Luc; 100 pg/embryo), renilla luciferase reporter (10 pg/embryo), and increasing amounts (0, 50, and 100 pg/embryo) of TcpAh expression vectors with stimulation of **(A)** Pam3CSK4 (200 pg/embryo), **(B)** CpG-ODN (400 pg/embryo), and **(C)** TNFα (10 pg/embryo) for 12 h. Data are the average luciferase activity ± SD (*p < 0.05; **p < 0.01; ***p < 0.001; ns, not significant). **(D)** Activation of the NF-κB-binding promoter detected in HEK293T cells transfected with NF-κB luciferase reporter (NF-κB-Luc; 150 ng/mL), renilla luciferase reporter (15 ng/mL), MyD88 expression vector (50 ng/mL), and increasing amounts (0, 100, and 500 ng/mL) of TcpAh expression vectors. Data are the average luciferase activity ± SD (**p < 0.01; ***p < 0.001). **(E,F)** Real-time PCR analysis for the expression of zebrafish IL-1β **(E)** and TNFα **(F)** in leukocytes, which were sorted from peripheral blood, spleen, and kidney tissues at indicated time after i.p. stimulation with PBS, wild-type *A. hydrophila* and Δ*tcpAh* mutant. Data are representative of three independent experiments as mean ± SD (**p < 0.01). Standard loading was indicated by β-actin expression.

Given that the TIR domains of TcpAh and zebrafish TLRs and adaptor proteins have structural homology, we propose that the potential regulatory function of TcpAh in zebrafish TLR signaling pathways is exerted through TIR–TIR homotypic interaction. An *in vivo* functional examination was performed using zebrafish embryo as a model to test this hypothesis. Zebrafish embryo was chosen because of its constitutive expression of various TLR signaling components during early development (2–24 hpf). For this procedure, one-cell-stage embryos were microinjected with NF-κB reporter vector in combination with different amounts of TcpAh-encoding plasmid and the stimulants Pam3CSK4, CpG-ODN and TNF-α for 12 h. As expected, the administration of TcpAh-encoding plasmid remarkably inhibited Pam3CSK4- and CpG-ODN-induced NF-κB activation in a dose-dependent manner (Figures 3A,B). By contrast, minimal alteration in NF-κB activity was detected in embryos exposed to TNF-α, whose signaling pathway is independent of TLRs and MyD88 (Figure 3C). These results indicated that TcpAh has a strong inhibitory effect on TLR signaling pathways, such as Pam3CSK4-induced TLR2 and CpG-ODN-induced TLR9 signaling pathways in this case, partially by association with MyD88 adaptor protein. Accordingly, TcpAh overexpression in HEK293T cells remarkably suppressed MyD88-induced NF-κB activation in a dose-dependent manner (Figure 3D). Furthermore, the expression of IL-1β and TNFα (two typical proinflammatory cytokines regulated by NF-κB signaling) in leukocytes was also dramatically impaired in wild-type *A. hydrophila*-infected zebrafish groups in comparison with those of Δ*tcpAh* mutant-infected groups (Figures 3E,F).

**FIGURE 4:**
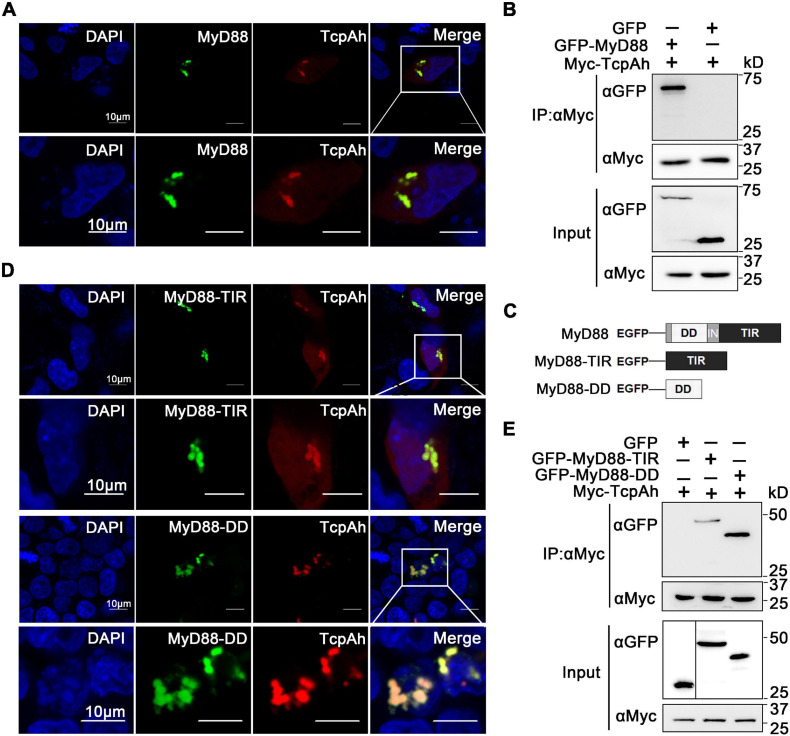
Interaction between TcpAh and MyD88 by TIR–TIR and TIR–DD interactions. **(A)** Co-localization analysis of TcpAh and zebrafish MyD88 proteins in HEK293T cells by confocal microscopy (Zeiss LSM 710; original magnification, 630×). The nucleus was stained with DAPI. Scale bars correspond to 10 μm. **(B)** Co-IP analysis between TcpAh and zebrafish MyD88 proteins from HEK293T cells expressing Myc-TcpAh with GFP or GFP-MyD88. **(C)** Schematic diagram of wild-type and domain-truncated zebrafish MyD88 forms. **(D)** Co-localization analysis between TcpAh and truncated MyD88 proteins in HEK293T cells by confocal microscopy (Zeiss LSM 710; original magnification, 630×). The nucleus was stained with DAPI. Scale bars correspond to 10 μm. **(E)** Co-IP assay between TcpAh and MyD88-TIR domain (148–284 aa) or MyD88-DD domain (11–101 aa) as shown in **(B)**, except MyD88-TIR or MyD88-DD was used instead of full-length wild-type MyD88.

Next, the association of TcpAh with MyD88 was examined by the intracellular co-localization of RFP-TcpAh and EGFP-MyD88 proteins in resting HEK293T cells or TLR-stimulated HEK293T cells and evidenced by the co-immunoprecipitation (Co-IP) between EGFP-MyD88 and Myc-TcpAh proteins (Figures 4A,B; Supplementary Figure 1A). To examine which domains in MyD88 were potentially involved in the interaction with TcpAh, EGFP-tagged MyD88 DD (11–101 aa) or TIR domain (148–284 aa) expression vectors were generated and co-transfected with RFP-tagged TcpAh in HEK293T cells (Figure 4C). The results showed that the two MyD88 mutants displayed strong co-localization with TcpAh; hence, DD and TIR domain contributed to the TcpAh–MyD88 interaction (Figure 4D). This notion was confirmed by Co-IP assay (Figure 4E). Previous studies have shown that bacterial TIR domain proteins, such as TcpB and TcpC, are associated with MyD88 through TIR–TIR interaction. Our present study showed that TcpAh can also associate with MyD88 by TIR–DD interaction in addition to TIR–TIR interaction.

### TcpAh Inhibits TRIF Signaling Pathway

**FIGURE 5:**
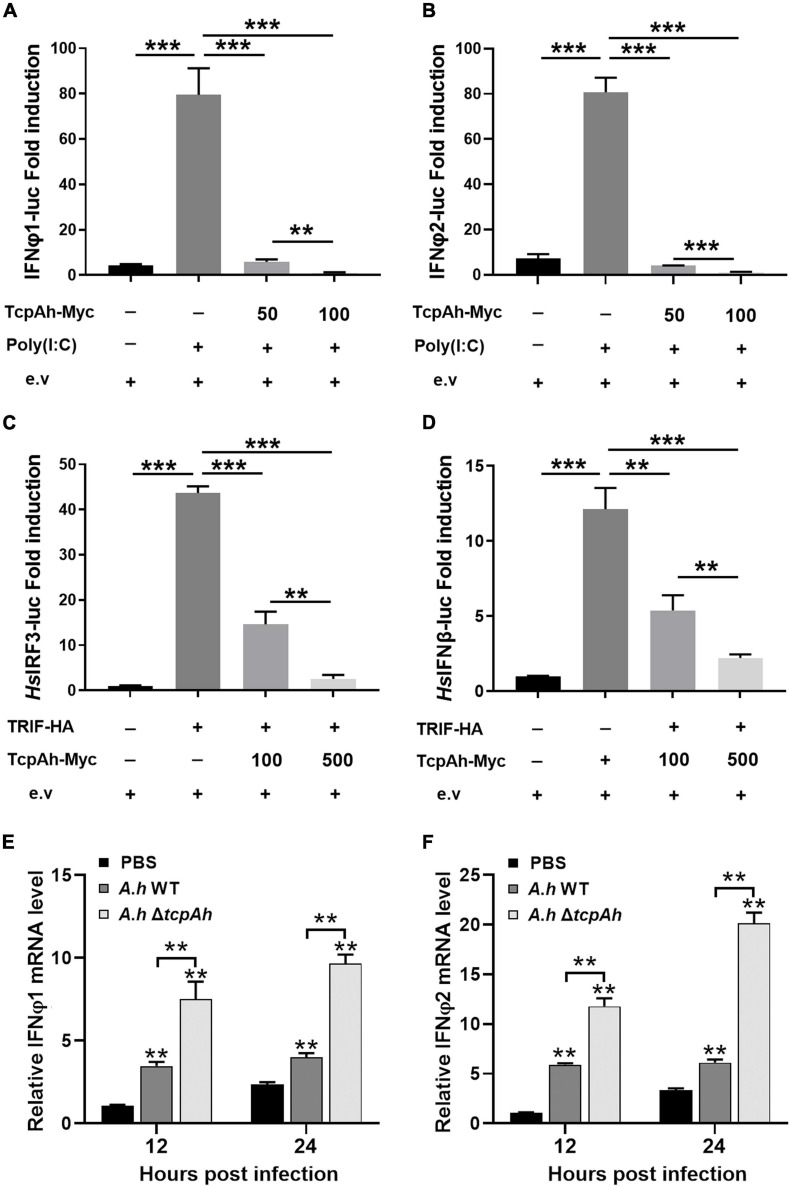
Examination of the inhibitory role of TcpAh in TRIF signaling pathway. **(A,B)** Activation of zebrafish IFNφ1/IFNφ2 promoters detected in zebrafish embryos microinjected with IFNφ1 or IFNφ2 luciferase reporter (IFNφ1 or IFNφ2-Luc; 100 pg/embryo), renilla luciferase reporter (10 pg/embryo), and increasing amounts (0, 50, and 100 pg/embryo) of TcpAh expression vectors under stimulation with PolyI:C (200 pg/embryo) for 12 h. Data are the average luciferase activity ± SD (**p < 0.01; ***p < 0.001). **(C,D)** Activation of human IRF3 and IFN-β promoters in HEK293T cells transfected with human IRF3 or IFN-β luciferase reporter (IRF3-Luc or IFN-β-Luc; 200 ng/mL), renilla luciferase reporter (15 ng/mL), zebrafish TRIF expression vector (50 ng/mL), and increasing amounts (0, 100, and 500 ng/mL) of TcpAh expression vectors. Data are the average luciferase activity ± SD (**p < 0.01; ***p < 0.001). **(E,F)** Real-time PCR analysis for the expression of zebrafish IFNφ1 **(E)** and IFNφ2 **(F)** in leukocytes, which were sorted from peripheral blood, spleen, and kidney tissues at indicated time after i.p. stimulation with PBS, wild-type *A. hydrophila* and Δ*tcpAh* mutant. Data are representative of three independent experiments as mean ± SD (**p < 0.01). Standard loading was indicated by β-actin expression.

Endocytosed TLRs, such as TLR3 and TLR4, trigger type-I IFN response in a TRIF-dependent manner. Recently, a TcpC protein from *E*. *coli* CFT073 was found to negatively regulate TRIF-dependent TLR signaling (Yadav et al., 2010). Zebrafish embryos were co-injected with an IFNφ1/φ2 (two typical type I interferon molecules in zebrafish) reporter vector and different amounts of TcpAh-encoding plasmid in combination with TLR3 agonist (PolyI:C) for 12 h to examine the potential role of TcpAh in TLR/TRIF-signaling axis. Results showed that TcpAh remarkably inhibited IFNφ1/φ2-reporter response to PolyI:C stimulation (Figures 5A,B). Similar results were also observed in HEK293T cells co-transfected with IRF3 or IFN-β reporter vector and zebrafish TRIF expression vector, and a mounting amount of TcpAh-Myc-encoding plasmid (Figures 5C,D). Furthermore, the expression of IFNφ1 and IFNφ2 in leukocytes was also dramatically impaired in wild-type *A. hydrophila*-infected zebrafish groups in comparison with those of Δ*tcpAh* mutant-infected groups (Figures 5E,F). These observations showed the ability of TcpAh to impair TRIF-mediated IFN signaling.

**FIGURE 6:**
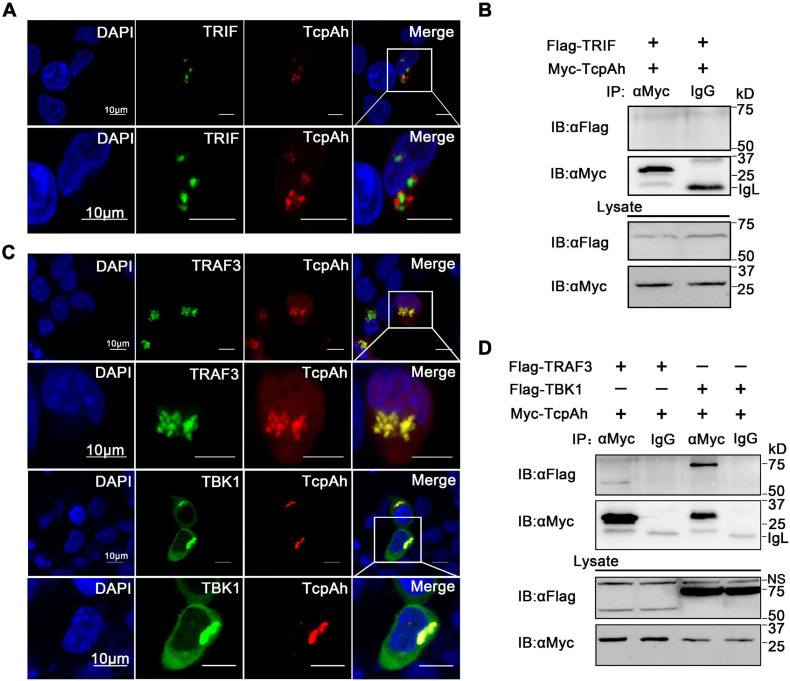
Examination of the associations of TcpAh with TRAF3 and TBK1. **(A)** Co-localization analysis of TcpAh and zebrafish TRIF protein in HEK293T cells by confocal microscopy (Zeiss LSM 710; original magnification, 630×). The nucleus was stained with DAPI. Scale bars correspond to 10 μm. **(B)** Co-IP analysis between TcpAh and zebrafish TRIF proteins from HEK293T cells expressing Myc-TcpAh with Flag-TRIF. **(C)** Co-localization analysis of TcpAh and zebrafish TRAF3 or TBK1 proteins in HEK293T cells by confocal microscopy (Zeiss LSM 710; original magnification, 630×). The nucleus was stained with DAPI. Scale bars correspond to 10 μm. **(D)** Co-IP analysis between TcpAh and zebrafish TRAF3 or TBK1 proteins from HEK293T cells expressing Myc-TcpAh with Flag-TRAF3 or Flag-TBK1.

**FIGURE 7:**
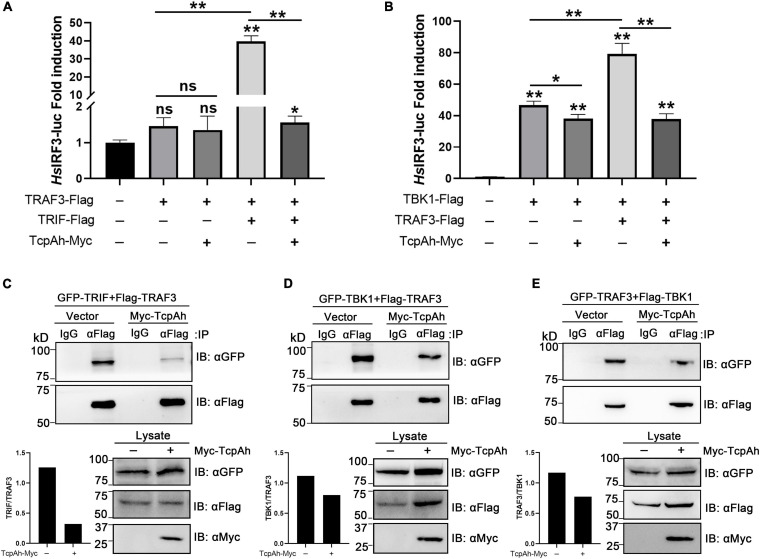
Examination of the functional role of TcpAh in preventing TRIF–TRAF3 and TRAF3–TBK1 interactions. **(A,B)** Activation of human IRF3 promoter in HEK293T cells transfected with human IRF3 luciferase reporter (*Hs*IRF3-Luc; 200 ng/mL), renilla luciferase reporter (15 ng/mL), and **(A)** zebrafish TRAF3 expression vector (50 ng/mL) alone or in combination with zebrafish TRIF expression vector (50 ng/mL) with or without TcpAh expression vector (500 ng/mL) or **(B)** zebrafish TBK1 expression vector (50 ng/mL) alone or in combination with a zebrafish TRAF3 expression vector (50 ng/mL) with or without TcpAh expression vector (500 ng/mL). Data are the average luciferase activity ± SD (*p < 0.05; **p < 0.01; ns, not significant). **(C–E)** Co-IP analysis showing the inhibition of TRIF–TRAF3 and TRAF3–TBK1 interactions by TcpAh in HEK293T cells transfected with plasmids in indicated combinations.

Given that TRIF is another TIR domain-containing protein that is potentially targeted by TcpAh; thus, an intracellular co-localization assay was performed to clarify this issue. Unexpectedly, minimal co-localization signal was detected between RFP-TcpAh and EGFP-TRIF by fluorescence imaging in resting HEK293T cells or TLR-stimulated HEK293T cells (Figure 6A; Supplementary Figure 1B), and no any interaction between Myc-TcpAh and Flag-TRIF was detected by Co-IP assay (Figure 6B). These results suggested that the ability of TcpAh to inhibit TRIF-mediated IFN signaling pathway was not by directly targeting TRIF itself, and was probably by association with TRAF3 and TBK1 downstream of the TRIF-mediated pathway. Expectedly, RFP-TcpAh was clearly co-localized with EGFP-TRAF3 or EGFP-TBK1 in resting HEK293T cells or TLR-stimulated HEK293T cells (Figure 6C; Supplementary Figure 1B). The associations of TcpAh with TRAF3 and TBK1 were further confirmed by Co-IP (Figure 6D). These data indicated that TcpAh inhibits the TRIF-signaling pathway through association with TRAF3 and TBK1. Next, we determined how TcpAh inhibits the TRIF-mediated IFN signaling pathway via association with TRAF3 and TBK1 through an IRF3 reporter assay. TRIF, TRAF3, TBK1, and TcpAh expression plasmids were co-transfected with IRF3 reporter vector into HEK293T cells in different combinations. The results showed that the co-transfection of TRIF and TRAF3, as well as the co-transfection of TBK1 and TRAF3, remarkably induced IRF3 promoter activity, and these reactions were remarkably inhibited in cells with TcpAh overexpression (Figures 7A,B). The outcomes suggested that TcpAh attenuated the TRIF-mediated signaling pathway by impairing TRIF–TRAF3 and TRAF3–TBK1 interactions. In support of this hypothesis, Co-IP assay clearly showed that the TRIF–TRAF3 and TRAF3–TBK1 interactions were both impaired by the intervention of TcpAh, and the TRIF–TRAF3 interaction was impaired much more seriously (Figures 7C–E). Altogether, TcpAh restrains TRIF-mediated signaling pathway by competitively interaction with TRAF3 and TBK1.

### Inhibition of TcpAh in Host Defense Against Infection

**FIGURE 8:**
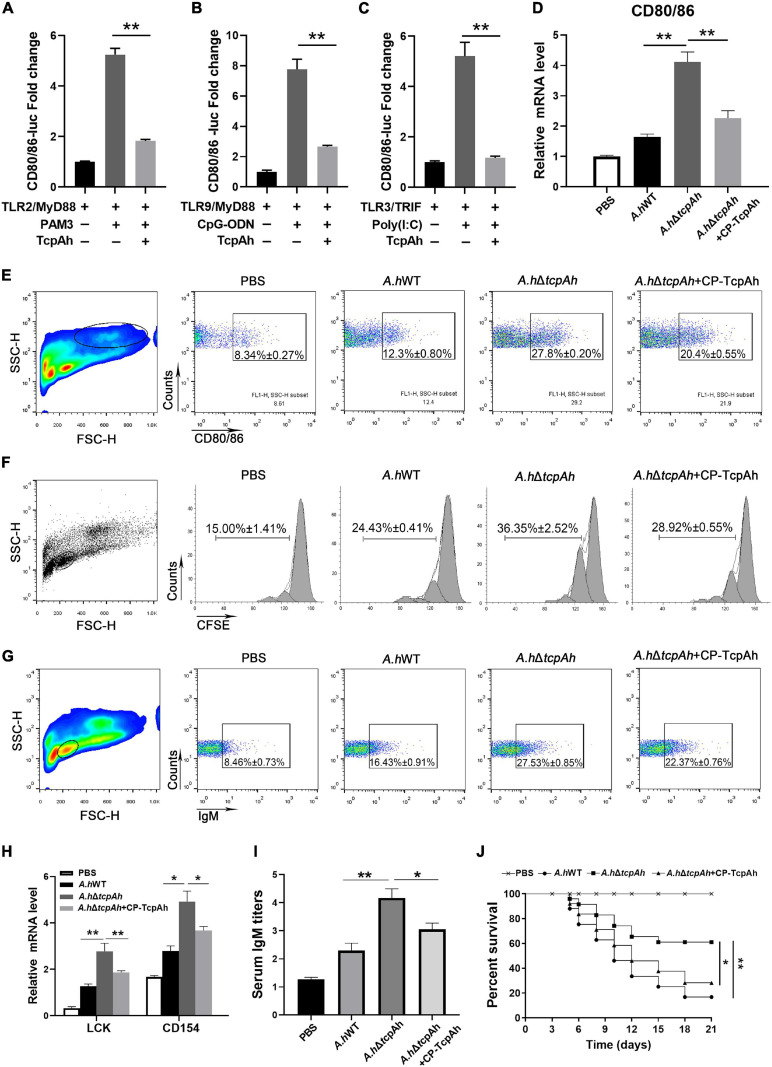
Examination of the inhibitory role of TcpAh in zebrafish adaptive humoral immunity against infection by repressing CD80/86 expression. **(A–C)** Activation of zebrafish CD80/86 promoter in HEK293T cells transfected with CD80/86 luciferase reporter (CD80/86-Luc; 200 ng/mL), renilla luciferase reporter (15 ng/mL) and expression vectors for zebrafish TLR2 or TLR9 or TLR3 (20 ng/mL) in combination with MyD88 or TRIF (20 ng/mL) with or without TcpAh expression vector (250 ng/mL). After 24 h, the HEK293T cells were stimulated with PAM3 or CpG-ODN or Poly(I:C) for 12 h. Data are the average luciferase activity ± SD (**p < 0.01; ***p < 0.001). **(D)** Real-time PCR analysis for the expression of zebrafish *cd80/86* in leukocytes, which were sorted from peripheral blood, spleen, and kidney tissues 2 days after i.p. stimulation with PBS, wild-type *A. hydrophila*, Δ*tcpAh* mutant, or Δ*tcpAh* mutant complemented with CP-TcpAh protein. Data are representative of three independent experiments as mean ± SD (*p < 0.05; **p < 0.01). **(E)** Flow cytometric analysis of CD80/86 expression level on MHC-II^+^ antigen-presenting cells (APCs) of each *in vivo* treatment group. Data are representative of three independent experiments as mean ± SD (*p < 0.05; **p < 0.01). **(F)** Proliferation of lymphocytes determined by CFSE dilution through flow cytometry under the indicated experimental treatment. **(G)** Proliferation of IgM^+^ B cells determined by flow cytometry under the indicated experimental treatment. Data are representative of three independent experiments as mean ± SD (*p < 0.05; **p < 0.01). **(H)** Real-time PCR analysis of the expression levels of zebrafish LCK and CD154 of each *in vitro* treatment group. Data are representative of three independent experiments as mean ± SD (*p < 0.05; **p < 0.01). **(I)** Examination of the inhibitory role of TcpAh in IgM production in response to *A*. *hydrophila* infection in each treatment group by ELISA. Data are representative of three independent experiments as mean ± SD (n = 20; *p < 0.05; **p < 0.01). **(J)** Examination of the inhibitory role of TcpAh in zebrafish defense against *A*. *hydrophila* infection. Zebrafish were infected with *A*. *hydrophila* Δ*tcpAh* or with wild-type *A*. *hydrophila* or with *A*. *hydrophila* Δ*tcpAh* complement with CP-TcpAh. Differences were analyzed using log-rank test (*p < 0.05; **p < 0.01). Group of fish injected with mock PBS was used as a negative control.

As *A*. *hydrophila* TcpAh down-modulates TLR signaling activation, we next sought to investigate whether TcpAh could interfere with zebrafish adaptive immunity. CD80/86 reporter assay clearly revealed that TcpAh suppressed the promotor activity of *cd80/86* gene by TLR2 or TLR9 or TLR3 signaling in HEK293T cells (Figures 8A–C). In addition, real-time PCR and flow cytometry analysis showed that zebrafish infected with Δ*tcpAh*-mutant *A*. *hydrophila* induced higher CD80/86 expression than those infected with wild-type strain at mRNA and protein levels (Figures 8D,E). This outcome suggested that TcpAh plays an inhibitory role in the expression of CD80/86 that is crucial for initiating adaptive immunity. We generated a cell-penetrating form of TcpAh (CP-TcpAh) with an appendant peptide for cell penetration at C-terminus to clarify this notion (Supplementary Figure 2A). This CP-TcpAh protein strongly inhibited NF-κB activity in HEK239T cells in a dose-dependent manner (Supplementary Figure 2B). The enhancement of CD80/86 expression induced by Δ*tcpAh* mutant in zebrafish was remarkably attenuated by the addition of CP-TcpAh protein (Figures 8D,E). Functionally, the Δ*tcpAh* mutant induced stronger T cell activation than wild-type *A*. *hydrophila in vitro* as determined by the higher cellular proliferation and transcriptional expression of LCK and CD154 in T cells upon Δ*tcpAh* mutant stimulation (Figures 8F,H). In this case, the enhanced T cell activation in response to Δ*tcpAh* mutant infection can be impaired by the restoration of CP-TcpAh (Figures 8F,H). The results implied that TcpAh inhibits the initiation of T cell activation by repressing CD80/86 expression on antigen-presenting cells, which potentially leads to the suppression of adaptive immunity against *A*. *hydrophila* infection. Thus, Ag-stimulated B cell activation and antibody (IgM) production were examined *in vivo*. Flow cytometric analysis showed that the percentage of B cells in Δ*tcpAh* mutant-administered groups (27.53% ± 0.85%) was dramatically upregulated (*p* < 0.01) in comparison with those of wild-type strain-infected groups (16.43% ± 0.91%) and mock PBS-treated control groups (8.46% ± 0.73%). The increased percentage of B cells in Δ*tcpAh* mutant-induced groups was remarkably decreased by administering CP-TcpAh protein (Figure 8G). Similar results were also detected in IgM production in various groups (Figure 8I). Furthermore, lethality declined in Δ*tcpAh*-mutant *A*. *hydrophila*-challenged zebrafish compared with that of the wild-type *A*. *hydrophila*-infected fish as shown by the survival rate increased from 20.0% to 64.0% (*p* < 0.01, log-rank test). The attenuated lethality of Δ*tcpAh*-mutant *A*. *hydrophila* was restored by the administration of CP-TcpAh protein in zebrafish and accompanied by the survival rate decreased from 64.0% to 32.0% (Figure 8J). The results suggested the inhibitory role of TcpAh in fish adaptive immune defense against infection and therefore uncovered a previously unrecognized strategy of *A*. *hydrophila* for immune evasion.

## Discussion

*Aeromonas hydrophila* is a group of Gram-negative bacteria that is widely distributed in aquatic environments (Lin et al., 2017). They are the causative agent of motile aeromonad septicemia (MAS) for a broad spectrum of host organisms, including mammals, amphibians, reptiles, and fish (Vivas et al., 2004). *A*. *hydrophila* causes disease outbreaks with high mortality in fish in aquaculture farms and severe economic losses to the aquaculture industry worldwide (Vivas et al., 2004; Jiang et al., 2017). Importantly, interest in the pathogenesis of *Aeromonas* now extends beyond the economic consequences to the fish farming industry, as members of this genus are increasingly implicated in intestinal and extraintestinal infections in humans (Thornley et al., 1997; Daskalov, 2006). *A*. *hydrophila* produces multiple virulence factors, including surface polysaccharides (such as capsule polysaccharide, lipopolysaccharide, and glucan), S-layers, iron-binding components, exotoxins, extracellular hydrolase, secretion complexes, fimbriae and other non-filamentous adhesins and flagella, which make the pathogenicity of this genus more complex (Alperi and Figueras, 2010; Tomas, 2012).

Although *A*. *hydrophila* possesses a variety of finely tuned pathogenic strategies that interfere with host defense against infection, TIR domain protein-mediated pathogenesis has not been reported in this species. In this study, we identified a TIR domain protein, namely TcpAh, from *A*. *hydrophila* JBN2301 strain, which is homologous to the TIR domains of TLRs. TcpAh ensures the efficient blockade of zebrafish immunity by interacting with MyD88, TRAF3, and TBK1, which are the three key components of TLR-mediated signaling pathways. Challenge assay showed that *tcpAh* deletion mutation substantially attenuated the virulence of *A*. *hydrophila* JBN2301 strain in zebrafish. Thus, TcpAh is a crucial virulence factor required for *A*. *hydrophila* infection. Importantly, nine additional highly virulent *A*. *hydrophila* strains were also predicted to contain such a TcpAh homolog. This finding suggests that TcpAh homologs ubiquitously exist in different *A*. *hydrophila* species. We believe that this study is the first to report the existence of a TIR domain protein-mediated pathogenic factor in *A*. *hydrophila*. Hence, this study uncovered a previously unrecognized virulence factor in this genus. TIR domain homologs have been found in many bacteria; these homologs include TlpA in *S*. *enterica*, TcpB in *B*. *melitensis*, TcpC in *E*. *coli*, YpTdp in *Y*. *pestis*, TirS in *S*. *aureus*, and PumA in *P*. *aeruginosa* (Newman et al., 2006; Cirl et al., 2008; Rana et al., 2011; Askarian et al., 2014; Imbert et al., 2017). Mechanistically, most of these TIR domain proteins function as inhibitors of host innate immunity by association with MyD88 or TIRAP through TIR–TIR interaction even though their molecular mode of action remains elusive (Rana et al., 2013). For example, *B*. *melitensis* TcpB, described as a molecular mimicry of TIRAP, can bind with MyD88 and TIRAP via TIR–TIR interaction to promote the ubiquitin-mediated degradation of TIRAP during bacterial infection (Cirl et al., 2008; Radhakrishnan et al., 2009). Our study showed that TcpAh can also interact with MyD88 through TIR–TIR interaction. More importantly, TIR–DD interaction, as an alternative binding manner, was utilized by TcpAh to associate with MyD88; thus, TcpAh may have a stronger binding activity to MyD88 than other TIR domain proteins by possessing additional TIR–DD interaction. This unconventional interaction also exists between TcpB and MyD88 (Chaudhary et al., 2012). Hence, the interaction between bacterial TIR-domain proteins and their targets are not always restricted to TIR–TIR interaction. Remarkably, we found that TcpAh can also associate with TRAF3 and TBK1, which are two critical components downstream of TRIF-dependent TLR signaling pathway. We believe that this study is the first to show that TRAF3 and TBK1 are the cellular targets of a bacterial TIR domain protein. This finding indicated that TcpAh has a broad spectrum of target proteins in innate immune signaling pathways in addition to its interaction with MyD88 to interfere with MyD88-dependent TLR signaling. The multiple associations of TcpAh with MyD88, TRAF3, and TBK1 endow this TIR domain protein a powerful pathogenic activity in the inhibition of host immunity against *A*. *hydrophila* infection at extensive levels, including MyD88- and TRIF-dependent immune responses. However, the precise mechanisms underlying the associations among TcpAh, MyD88, TRAF3 and TBK1 independent of the canonical TIR–TIR interaction remain to be further clarified. Additionally, the current interpretation of the intervention of bacterial TIR domain proteins to host immune defense largely focused on innate immunity; whether bacterial TIR domain proteins have an influence on host adaptive immunity remains poorly understood. In the present study, we found that TcpAh plays an inhibitory role in adaptive humoral immunity against *A*. *hydrophila* infection in zebrafish as determined by an enhanced activation of T and B cells and an increased production of IgM Abs in fish upon Δ*tcpAh* mutant infection. This enhancement can be impaired by administering a recombinant TcpAh protein tagged with a cell penetration peptide (CP-TcpAh). In addition, TcpAh remarkably suppressed CD80/86 activation by attenuating NF-κB signaling as examined by an *in vitro* reporter assay. Infection with Δ*tcpAh* mutant *A*. *hydrophila* dramatically upregulated CD80/86 expression in fish, and this outcome was attenuated by supplementing CP-TcpAh protein. These observations suggested that TcpAh plays an inhibitory role in adaptive humoral immunity through the attenuation of CD80/86 costimulatory signal, which is an important connection between innate and adaptive immunities and a crucial initiator of the activation of adaptive immunity.

In conclusion, our study identified a TIR domain protein (TcpAh) from *A*. *hydrophila*, which can be considered a new member of the bacterial TIR domain protein family. TcpAh acts as a strong virulence effector by extensively targeting MyD88, TRAF3, and TBK1 downstream of TLR signaling pathways, which leads to the inhibition of CD80/86 costimulatory signal essential for the activation of adaptive immunity. Thus, our findings uncovered a previously unrecognized mechanism underlying *A*. *hydrophila* evades from host immune defense, which will benefit the development of therapeutic interventions for *A*. *hydrophila* infection. Particularly, TcpAh can become a promising target for drug therapy because of its great potential use in *A*. *hydrophila*-elicited diseases, such as motile aeromonad septicemia in fish and other species.

## Data Availability Statement

The raw data supporting the conclusions of this article will be made available by the authors, without undue reservation.

## Ethics Statement

The animal study was reviewed and approved by the Committee on Animal Care and Use and the Committee on the Ethic of Animal Experiments of Zhejiang University.

## Author Contributions

J-ZS, H-PT, and L-XX conceived and designed the experiments. H-PT, CH, HL, and C-BH performed the experiments. H-PT, CH, J-FJ, JB, TS, and A-FL analyzed the data. L-XX, D-DF, and J-ZS contributed reagents, materials, and analysis tools. H-PT, CH, and J-ZS wrote the manuscript. All authors contributed to the article and approved the submitted version.

## Conflict of Interest

The authors declare that the research was conducted in the absence of any commercial or financial relationships that could be construed as a potential conflict of interest.

## References

[B1] Akira S. Takeda K. (2004). Toll-like receptor signalling. *Nat. Rev. Immunol.* 4 499–511. 15229469 10.1038/nri1391

[B2] Alperi A. Figueras M. J. (2010). Human isolates of Aeromonas possess Shiga toxin genes (stx1 and stx2) highly similar to the most virulent gene variants of *Escherichia coli*. *Clin. Microbiol. Infec.* 16 1563–1567. 10.1111/j.1469-0691.2010.03203.x 20219084

[B3] Askarian F. van Sorge N. M. Sangvik M. Beasley F. C. Henriksen J. R. Sollid J. U. (2014). A Staphylococcus aureus TIR domain protein virulence factor blocks TLR2-mediated NF-kappaB signaling. *J. Innate Immun.* 6 485–498. 10.1159/000357618 24481289 PMC4198549

[B4] Burns K. Janssens S. Brissoni B. Olivos N. Beyaert R. Tschopp J. (2003). Inhibition of interleukin 1 receptor/toll-like receptor signaling through the alternatively spliced, short form of MyD88 is due to its failure to recruit IRAK-4. *J. Exp. Med.* 197 263–268. 10.1084/jem.20021790 12538665 PMC2193806

[B5] Carty M. Goodbody R. Schroder M. Stack J. Moynagh P. N. Bowie A. G. (2006). The human adaptor SARM negatively regulates adaptor protein TRIF-dependent Toll-like receptor signaling. *Nat. Immunol.* 7 1074–1081. 10.1038/ni1382 16964262

[B6] Chaudhary A. Ganguly K. Cabantous S. Waldo G. S. Micheva-Viteva S. N. Nag K. (2012). The Brucella TIR-like protein TcpB interacts with the death domain of MyD88. *Biochem. Biophys. Res. Commun.* 417 299–304. 10.1016/j.bbrc.2011.11.104 22155231 PMC3607435

[B7] Cirl C. Wieser A. Yadav M. Duerr S. Schubert S. Fischer H. (2008). Subversion of Toll-like receptor signaling by a unique family of bacterial Toll/interleukin-1 receptor domain-containing proteins. *Nat. Med.* 14 399–406. 10.1038/nm1734 18327267

[B8] Crotty S. (2011). Follicular helper CD4 T cells (TFH). *Annu. Rev. Immunol.* 29 621–663. 10.1146/annurev-immunol-031210-101400 21314428

[B9] Daskalov H. (2006). The importance of Aeromonas hydrophila in food safety. *Food Control.* 17 474–483. 10.1016/j.foodcont.2005.02.009

[B10] Dunne A. Ejdeback M. Ludidi P. L. O’Neill L. A. J. Gay N. J. (2003). Structural complementarity of Toll/interleukin-1 receptor domains in toll-like receptors and the adaptors Mal and MyD88. *J. Biol. Chem.* 278 41443–41451. 10.1074/jbc.m301742200 12888566

[B11] Dunne A. O’Neill L. A. (2003). The interleukin-1 receptor/Toll-like receptor superfamily: signal transduction during inflammation and host defense. *Sci. STKE* 2003:re3. 10.1126/stke.2003.171.re3 12606705

[B12] Fearns C. Pan Q. Mathison J. C. Chuang T. H. (2006). Triad3A regulates ubiquitination and proteasomal degradation of RIP1 following disruption of Hsp90 binding. *J. Biol. Chem.* 281 34592–34600. 10.1074/jbc.m604019200 16968706

[B13] Greenfield E. A. Nguyen K. A. Kuchroo V. K. (1998). CD28/B7 costimulation: a review. *Crit. Rev. Immunol.* 18 389–418. 10.1615/critrevimmunol.v18.i5.10 9784967

[B14] Harikrishnan R. Rani M. N. Balasundaram C. (2003). Hematological and biochemical parameters in common carp, Cyprinus carpio, following herbal treatment for Aeromonas hydrophila infection. *Aquaculture* 221 41–50. 10.1016/s0044-8486(03)00023-1

[B15] Horng T. Barton G. M. Flavell R. A. Medzhitov R. (2002). The adaptor molecule TIRAP provides signalling specificity for Toll-like receptors. *Nature* 420 329–333. 10.1038/nature01180 12447442

[B16] Imbert P. R. Louche A. Luizet J. B. Grandjean T. Bigot S. Wood T. E. (2017). A *Pseudomonas aeruginosa* TIR effector mediates immune evasion by targeting UBAP1 and TLR adaptors. *EMBO J.* 36 1869–1887. 10.15252/embj.201695343 28483816 PMC5494471

[B17] Janssens S. Beyaert R. (2002). A universal role for MyD88 in TLR/IL-1R-mediated signaling. *Trends Biochem. Sci.* 27 474–482. 10.1016/s0968-0004(02)02145-x 12217523

[B18] Ji J. Liao Z. Rao Y. Li W. Yang C. Yuan G. (2019). Thoroughly Remold the Localization and Signaling Pathway of TLR22. *Front. Immunol.* 10:3003. 10.3389/fimmu.2019.03003 eCollection 2019 32010127 PMC6978911

[B19] Jiang Q. L. Chen W. B. Qin Y. X. Huang L. X. Xu X. J. Zhao L. M. (2017). AcuC, a histone deacetylase, contributes to the pathogenicity of Aeromonas hydrophila. *Microbiologyopen* 6:e00468. 10.1002/mbo3.468 28371510 PMC5552924

[B20] Jin M. S. Lee J. O. (2008). Structures of the toll-like receptor family and its ligand complexes. *Immunity* 29 182–191. 10.1016/j.immuni.2008.07.007 18701082

[B21] Kobayashi K. Hernandez L. D. Galan J. E. Janeway C. A. Medzhitov R. Flavell R. A. (2002). IRAK-M is a negative regulator of toll-like receptor signaling. *Cell* 110 191–202. 10.1016/s0092-8674(02)00827-9 12150927

[B22] Lin G. F. Chen W. B. Su Y. Q. Qin Y. X. Huang L. X. Yan Q. P. (2017). Ribose operon repressor (RbsR) contributes to the adhesion of Aeromonas hydrophila to Anguilla japonica mucus. *Microbiologyopen* 6:e00451. 10.1002/mbo3.451 PMC5552941 28127946

[B23] Low L. Y. Mukasa T. Reed J. C. Pascual J. (2007). Characterization of a TIR-like protein from Paracoccus denitrificans. *Biochem. Biophys. Res. Commun.* 356 481–486. 10.1016/j.bbrc.2007.03.003 17362878 PMC1880877

[B24] Lu L. F. Li S. Lu X. B. LaPatra S. E. Zhang N. Zhang X. J. (2016). Spring Viremia of Carp Virus N Protein Suppresses Fish IFNphi1 Production by Targeting the Mitochondrial Antiviral Signaling Protein. *J. Immunol.* 196 3744–3753. 10.4049/jimmunol.1502038 26994222

[B25] Ma J. X. Li J. Y. Fan D. D. Feng W. Lin A. F. Xiang L. X. (2018). Identification of DEAD-Box RNA Helicase DDX41 as a Trafficking Protein That Involves in Multiple Innate Immune Signaling Pathways in a Zebrafish Model. *Front. Immunol.* 9:1327. 10.3389/fimmu.2018.01327 eCollection 2018 29942316 PMC6005158

[B26] Newman R. M. Salunkhe P. Godzik A. Reed J. C. (2006). Identification and characterization of a novel bacterial virulence factor that shares homology with mammalian Toll/interleukin-1 receptor family proteins. *Infect. Immun.* 74 594–601. 10.1128/iai.74.1.594-601.2006 16369016 PMC1346628

[B27] O’Neill L. A. (2003). SIGIRR puts the brakes on Toll-like receptors. *Nat. Immunol.* 4 823–824. 10.1038/ni0903-823 12942080

[B28] O’Neill L. A. J. Bowie A. G. (2007). The family of five: tIR-domain-containing adaptors in Toll-like receptor signalling. *Nat. Rev. Immunol.* 7 353–364. 10.1038/nri2079 17457343

[B29] Oshiumi H. Matsumoto M. Funami K. Akazawa T. Seya T. (2003). TICAM-1, an adaptor molecule that participates in Toll-like receptor 3-mediated interferon-beta induction. *Nat. Immunol.* 4 161–167. 10.1038/ni886 12539043

[B30] Pasare C. Medzhitov R. (2004). Toll-like receptors: linking innate and adaptive immunity. *Microb. Infect.* 6 1382–1387. 10.1016/j.micinf.2004.08.018 15596124

[B31] Poltorak A. He X. L. Smirnova I. Liu M. Y. Van Huffel C. Du X. (1998). Defective LPS signaling in C3H/HeJ and C57BL/10ScCr mice: mutations in Tlr4 gene. *Science* 282 2085–2088. 10.1126/science.282.5396.2085 9851930

[B32] Radhakrishnan G. K. Yu Q. Harms J. S. Splitter G. A. (2009). Brucella TIR Domain-containing Protein Mimics Properties of the Toll-like Receptor Adaptor Protein TIRAP. *J. Biol. Chem.* 284 9892–9898. 10.1074/jbc.m805458200 19196716 PMC2665112

[B33] Rana R. R. Simpson P. Zhang M. Jennions M. Ukegbu C. Spear A. M. (2011). Yersinia pestis TIR-domain protein forms dimers that interact with the human adaptor protein MyD88. *Microb. Pathog.* 51 89–95. 10.1016/j.micpath.2011.05.004 21640812

[B34] Rana R. R. Zhang M. Spear A. M. Atkins H. S. Byrne B. (2013). Bacterial TIR-containing proteins and host innate immune system evasion. *Med. Microbiol. Immunol.* 202 1–10. 10.1007/s00430-012-0253-2 22772799

[B35] Rojas M. Donahue J. P. Tan Z. J. Lin Y. Z. (1998). Genetic engineering of proteins with cell membrane permeability. *Nat. Biotechnol.* 16 370–375. 10.1038/nbt0498-370 9555729

[B36] Rosadini C. V. Kagan J. C. (2015). Microbial strategies for antagonizing Toll-like-receptor signal transduction. *Curr. Opinion Immunol.* 32 61–70. 10.1016/j.coi.2014.12.011 25615700 PMC4336813

[B37] Saraceni P. R. Romero A. Figueras A. Novoa B. (2016). Establishment of Infection Models in Zebrafish Larvae (Danio rerio) to Study the Pathogenesis of Aeromonas hydrophila. *Front. Microbiol.* 7:1219. 10.3389/fmicb.2016.01219 27540375 PMC4972827

[B38] Shao T. Shi W. Zheng J. Y. Xu X. X. Lin A. F. Xiang L. X. (2018). Costimulatory Function of Cd58/Cd2 Interaction in Adaptive Humoral Immunity in a Zebrafish Model. *Front. Immunol.* 9:1204. 10.3389/fimmu.2018.01204 eCollection 2018 29904386 PMC5990624

[B39] Slack J. L. Schooley K. Bonnert T. P. Mitcham J. L. Qwarnstrom E. E. Sims J. E. (2000). Identification of two major sites in the type I interleukin-1 receptor cytoplasmic region responsible for coupling to pro-inflammatory signaling pathways. *J. Biol. Chem.* 275 4670–4678. 10.1074/jbc.275.7.4670 10671496

[B40] Thornley J. P. Shaw J. G. Gryllos I. A. Eley A. (1997). Virulence properties of clinically significant Aeromonas species: evidence for pathogenicity. *Rev. Med. Microbiol.* 8 61–72. 10.1097/00013542-199704000-00002

[B41] Tobias P. S. Lee H. K. Orr S. Soldau K. Tapping R. (2000). Innate immune system recognition of microbial pathogens. *Immunol. Res.* 21 341–343. 10.1385/ir:21:2-3:341 10852135

[B42] Tomas J. M. (2012). The main Aeromonas pathogenic factors. *ISRN Microbiol.* 2012:256261. 10.5402/2012/256261 PMC3658858 23724321

[B43] Underhill D. M. Ozinsky A. Hajjar A. M. Stevens A. Wilson C. B. Bassetti M. (1999). The Toll-like receptor 2 is recruited to macrophage phagosomes and discriminates between pathogens. *Nature* 401 811–815. 10.1038/44605 10548109

[B44] Vivas J. Carracedo B. Riano J. Razquin B. E. Lopez-Fierro P. Acosta F. (2004). Behavior of an Aeromonas hydrophila aroA live vaccine in water microcosms. *Appl. Environ. Microb.* 70 2702–2708. 10.1128/aem.70.5.2702-2708.2004 15128521 PMC404459

[B45] Wald D. Qin J. Z. Zhao Z. D. Qian Y. C. Naramura M. Tian L. P. (2003). SIGIRR, a negative regulator of Toll-like receptor-interleukin 1 receptor signaling. *Nat. Immunol.* 4 920–927. 10.1038/ni968 12925853

[B46] Wan F. Hu C. B. Ma J. X. Gao K. Xiang L. X. Shao J. Z. (2016). Characterization of γδ T Cells from Zebrafish Provides Insights into Their Important Role in Adaptive Humoral Immunity. *Front. Immunol.* 7:675. 10.3389/fimmu.2016.00675 eCollection 2016 28119690 PMC5220103

[B47] Xie Q. Mei W. Ye X. Zhou P. Islam M. S. Elbassiony K. R. A. (2018). The two-component regulatory system CpxA/R is required for the pathogenesis of Aeromonas hydrophila. *FEMS Microbiol. Lett.* 1:365. 10.1093/femsle/fny218 30184189

[B48] Xu X. G. Hu J. F. Ma J. X. Nie L. Shao T. Xiang L. X. (2016). Essential Roles of TIM-1 and TIM-4 Homologs in Adaptive Humoral Immunity in a Zebrafish Model. *J Immunol* 196 1686–1699. 10.4049/jimmunol.1501736 26792807

[B49] Xu Y. W. Tao X. Shen B. H. Horng T. Medzhitov R. Manley J. L. (2000). Structural basis for signal transduction by the Toll/interleukin-1 receptor domains. *Nature* 408 111–115. 10.1038/35040600 11081518

[B50] Yadav M. Zhang J. Fischer H. Huang W. Lutay N. Cirl C. (2010). Inhibition of TIR domain signaling by TcpC: myD88-dependent and independent effects on *Escherichia coli* virulence. *PLoS Pathog.* 6:e1001120. 10.1371/journal.ppat.1001120 20886104 PMC2944809

[B51] Yamamoto M. Sato S. Hemmi H. Hoshino K. Kaisho T. Sanjo H. (2003a). Role of adaptor TRIF in the MyD88-independent toll-like receptor signaling pathway. *Science* 301 640–643. 10.1126/science.1087262 12855817

[B52] Yamamoto M. Sato S. Hemmi H. Uematsu S. Hoshino K. Kaisho T. (2003b). TRAM is specifically involved in the Toll-like receptor 4-mediated MyD88-independent signaling pathway. *Nat. Immunol.* 4 1144–1150. 10.1038/ni986 14556004

[B53] Yamamoto M. Sato S. Mori K. Hoshino K. Takeuchi O. Takeda K. (2002). Cutting edge: a novel toll/IL-1 receptor Domain containing adapter that preferentially activates the IFN-beta promoter in the toll-like receptor signaling. *J. Immunol.* 169 6668–6672. 10.4049/jimmunol.169.12.6668 12471095

