# Supplementary material for: Inhibitory Role of an Aeromonas hydrophila TIR Domain Effector in Antibacterial Immunity by Targeting TLR Signaling Complexes in Zebrafish
Source: Front Microbiol. 2021 Jul 8;12:694081. doi: 10.3389/fmicb.2021.694081 (PMC8297594; doi:10.3389/fmicb.2021.694081)
Supplement: Supplementary file 1 [file Data_Sheet_1.pdf]

Supplementary Material

Supplementary Table 1
Strain and accession numbers and localization of *tcpAh* in genome of *Aeromonas hydrophila* (NCBI)

**Strain no.**	**Accession no.**	**Localization**
JBN2301	CP013178.1	AS145_07865
ZYAH72	CP016989.1	BFW97_07865
D4	CP013965.1	AhyD4_07830
LHW39	CP050012.1	G9455_08240
NJ-35	CP006870.1	Between U876_07580 and U876_07590
J-1	CP006883.1	Between V469_07605 and V469_07620
pc104A	CP007576.1	Between V429_15525 and V429_15545
AL09-71	CP007566.1	Between V428_15495 and V428_15510
ML09-119	CP005966.1	Between AHML_14990 and AHML_14995
GYK1	CP016392.1	Between A9258_07570 and A9258_07575
