# Supplementary material for: Inhibitory Role of an Aeromonas hydrophila TIR Domain Effector in Antibacterial Immunity by Targeting TLR Signaling Complexes in Zebrafish
Source: Front Microbiol. 2021 Jul 8;12:694081. doi: 10.3389/fmicb.2021.694081 (PMC8297594; doi:10.3389/fmicb.2021.694081)
Supplement: Supplementary file 2 [file Data_Sheet_2.pdf]

Supplementary Table 2
List of bacterial strains, plasmids and primers used in this study

Bacterial strains/plasmids	Description	Source
E. coli SMλpir	Host for carrying the recombinant plasmids	Purchased
A. hydrophila JBN2301	Pathogenic WT	Purchased
Δ*tcpAh*	JBN2301 derivative, *tcpAh* deletion mutant	This work
pRE112	Suicide vector, sacBmobRP4 R6k ori, Cm^r	Purchased
pRE112-Δ*tcpAh*Tc^r	pRE112 derivative, designed for knockout of *tcpAh*, Cm^r, Tc^r	This work
pBBR1MCS-3	Donor of Tc^r gene cassette for pRE112-Δ*tcpAh*	Purchased
**Primers Sequence (5'-3')**	**Product size (bp)**	**Target gene or Application**
P1 ctcgatatcgcatgcggtaccTGTTGAAGAATACG ATGACATTCCG<br>P2 CTTATCTCCCTATTGACTGCTTATTGC	1148	Upstream fragment
P3 GTGATTTTCTGGATGTATAGCTAAGGTG<br>P4 caagcttcttctagaggtaccACATACTGGACGGTT TAGCTGATG	1184	Downstream fragment
P5 GCAATAAGCAGTCAATAGGGAGATAAG TTGACAGCTTATCATCGATAAGCTTT<br>P6 CACCTTAGCTATACATCCAGAAAATCAC TCAGGTCGAGGTGGCCCG	1267	Tc^r cassette fragment
P7 ATCCTGCCTGGTTGTGCTTATC<br>P8 AGTTCGCTCCTGCGAGTCCCCT	1261(WT)/1949(Δ*tcpAh*)	Identification
tcpAh-F CCGGAATTCATGGCCTATTTTAC TAAAAGTGAGGC<br>*tcpAh*-R CCGCTCGAGTTATGATGAATAGC GATTCCACGATGG	579	Eukaryotic or Prokaryotic expression
tcpAh-F1 agtccggactcagatctcgagctATGGCCTAT TTTACTAAAAGTGAGGC<br>*tcpAh*-R1 ttatctagatccggtggatccTTATGATGAA TAGCGATTCCACGATGG	579	Eukaryotic expression
CP-*tcpAh*-F CCGGAATTCATGGCCTATTTTAC TAAAAGTGAGGC<br>CP-*tcpAh*-R CCGCTCGAGTTAGGGTGCGGCAAGAAG AACAGGGAGAAGAACGGCTGCTGATGA	579	Prokaryotic expression

ATAGCGATTCCAC		
MyD88-F ggcctagcgatcgcaaagcttATGGCATCA AAGTTAAGTCTAGACCA<br>*MyD88*-R gtatgggtaaccggtaagcttGGGCAGTGA GAGTGCTTTGG	852	Eukaryotic expression
MyD88-TIR-F gcctagcgatcgcaaagcttACCTTTGATGCTTTCA TCTGCTACT<br>*MyD88-TIR*-R gtatgggtaaccggtaagcttGGGCAGT GAGAGTGCTTTGG	411	Eukaryotic expression
MyD88-DD-F ggcctagcgatcgcaaagcttGCGATT CCAGTAACAGCTTTAAACT<br>*MyD88-DD*-R gtatgggtaaccggtaagcttAATTTCT TTGAGCTCGGATATGA	273	Eukaryotic expression
TRIF-F ggcctagcgatcgcaaagcttATGGCAGAAG GTGGAATGAAGCCTT<br>*TRIF*-R gtatgggtaaccggtaagcttCGACTCTTCGG CTGAGCTTTTAGTG	1698	Eukaryotic expression
TRIF-F1 tcccccgggctgcaggaattcGCCACCATGG CAGAAGGTGGAATGAAGCCTT<br>*TRIF*-R2 gataagcttgatatcgaattcCTACGACTCTT CGGCTGAGCTTTTAGTG	1698	Eukaryotic expression
TRAF3-F agtccggactcagatctcgagctATGTCCGCA GGGCGTAATG<br>*TRAF3*-R ttatctagatccggtggatccTCAAGGGTCAG GGAGGTCTGA	1722	Eukaryotic expression
TRAF3-F1 tcccccgggctgcaggaattcGCCACCATGT CCGCAGGGCGTAATG<br>*TRAF3*-R1 gataagcttgatatcgaattcTCAAGGGTCA GGGAGGTCTGA	1722	Eukaryotic expression
TBK1-F agtccggactcagatctcgagctATGCAGAGTA CGGCCAATTACC<br>*TBK1*-R ttatctagatccggtggatccTCACATCCGCTC CACTGTCCT	2184	Eukaryotic expression
TBK1-F1 tcccccgggctgcaggaattcGCCACCATGC AGAGTACGGCCAATTACC<br>*TBK1*-R1 gataagcttgatatcgaattcTCACATCCGCT CCACTGTCCT	2184	Eukaryotic expression
RFP-F tgaaccgtcagatccgctagcgctaccggtcgccaccAT GGCCTCCTCCGAGAACGT<br>*RFP*-R aattcgaagcttgagctcgagatctgagtccggaCAGG AACAGGTGGTGGCGG	675	ORF cloning

cd80/86-pGL-F：ACCAACAGTACCGGAATGCGCGTTCATCACCAGGCTAATA<br>*cd80/86*-pGL-R：CGAGATCTGCGATCTAAGTAAAGAAGGTGATGTCATGCC	2000	*cd80/86* promoter cloning
cd80/86-qF GCTGTGACATAGGAGGCTCTG<br>*cd80/86*-qR ACCTACTCCAAAGGAATGTGAGC	214	qRT-PCR
β-actin-qF AGGTCATCACCATCGGCAAT<br>*β-actin*-qR GATGTCCACGTCGCACTTCA	131	qRT-PCR
lck-qF CTCCTCTTCCAGAGAACCTGGT<br>*lck*-qR TCATGGCGTCATTCCTGGAG	244	qRT-PCR
cd154-qF AGTCAACGGGGAACTGCAATG<br>*cd154*-qR TCTGGACGCCTTGTAGCAGTG	308	qRT-PCR
DrIL-1β-qF TGGACTTCGCAGCACAAAATG<br>*DrIL-1β*-qR GTTCACTTCACGCTCTTGGATG	150	qRT-PCR
DrTNFα-qF GCTGGATCTTCAAAGTCGGGTGTA<br>*DrTNFα*-qR TCTCAGCACACTTCCATCTTGTTG	144	qRT-PCR
DrIFNφ1-qF TGGAGGACCAGGTGAAGTT<br>*DrIFNφ1*-qR ATTGACCCTTGCGTTGCTT	342	qRT-PCR
DrIFNφ2-qF TCTACTTGAGAGTATGGGCGGTC<br>*DrIFNφ2*-qR GTTCCTTGAGCTCTCATCCTCATA	276	qRT-PCR

F: Forward primer; R: Reverse primer.
