# Supplementary material for: Inhibitory Role of an Aeromonas hydrophila TIR Domain Effector in Antibacterial Immunity by Targeting TLR Signaling Complexes in Zebrafish
Source: Front Microbiol. 2021 Jul 8;12:694081. doi: 10.3389/fmicb.2021.694081 (PMC8297594; doi:10.3389/fmicb.2021.694081)
Supplement: Supplementary file 3 [file Image_1.pdf]

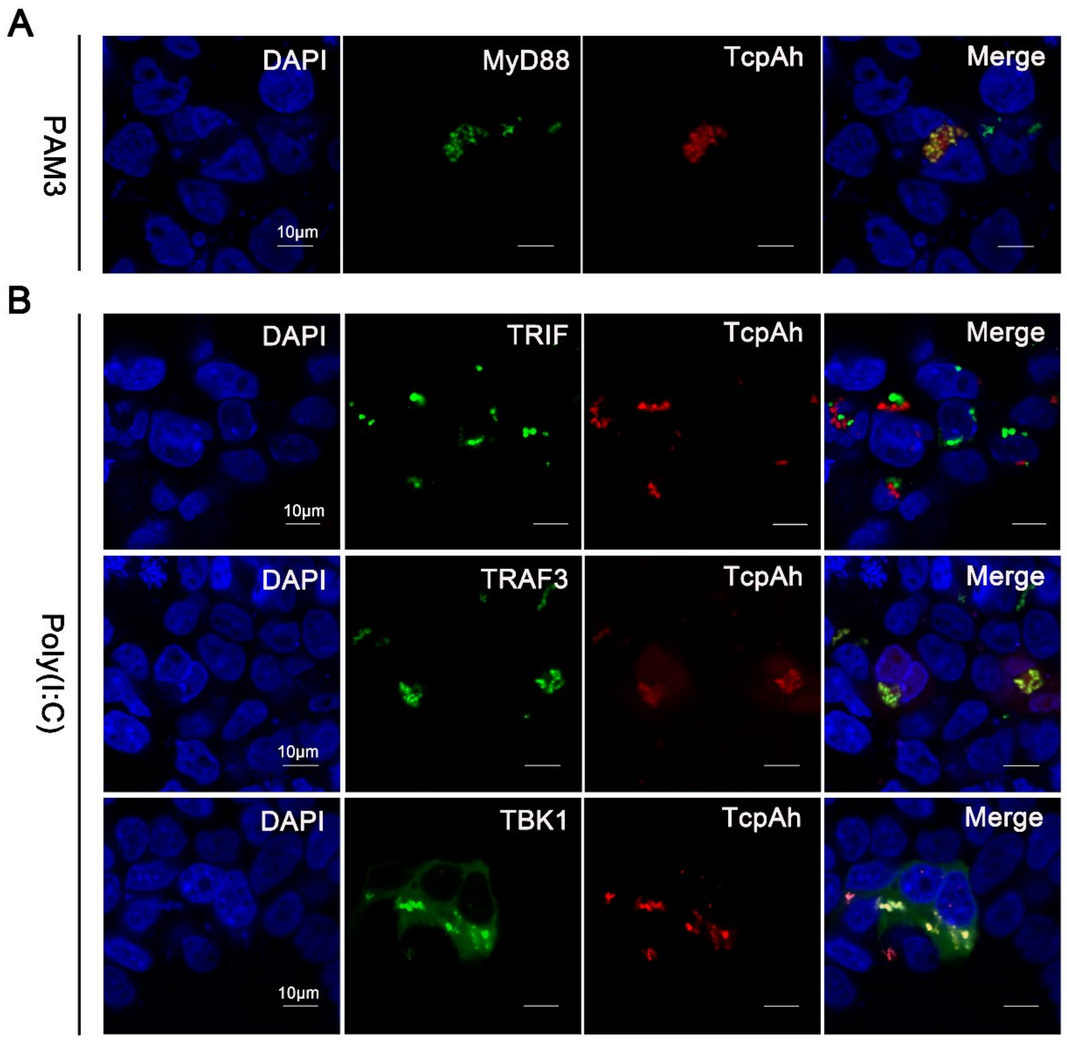

Supplementary Figure 1. (A) Co-localization analysis of TcpAh and zebrafish MyD88 protein in HEK293T cells upon Pam3CSK stimulation by confocal microscopy (Zeiss LSM 710; original magnification, 630×). The nucleus was stained with DAPI. Scale bars correspond to 10 μm. **(B)** Co-localization analysis of TcpAh and zebrafish TRIF, TRAF3 and TBK1 proteins in HEK293T cells upon Poly(I:C) stimulation by confocal microscopy (Zeiss LSM 710; original magnification, 630×). The nucleus was stained with DAPI. Scale bars correspond to 10 μm.
