# Supplementary material for: Inhibitory Role of an Aeromonas hydrophila TIR Domain Effector in Antibacterial Immunity by Targeting TLR Signaling Complexes in Zebrafish
Source: Front Microbiol. 2021 Jul 8;12:694081. doi: 10.3389/fmicb.2021.694081 (PMC8297594; doi:10.3389/fmicb.2021.694081)
Supplement: Supplementary file 4 [file Image_2.pdf]

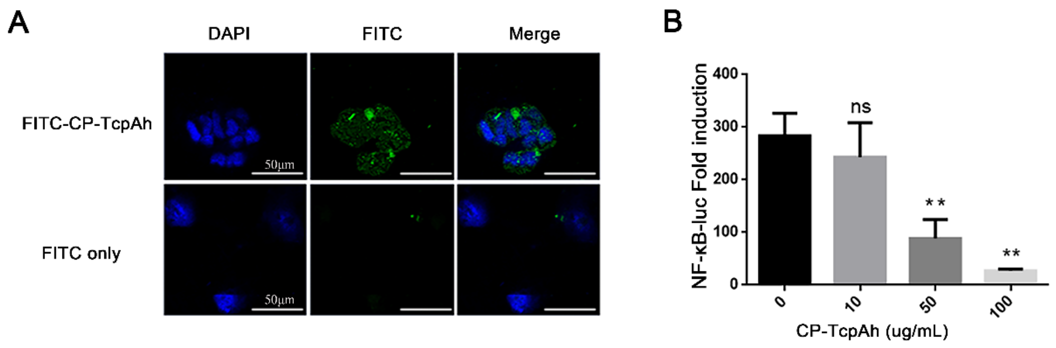

Supplementary Figure 2. (A) Examination of the penetrating ability of recombinant CP-TcpAh protein. The CP-TcpAh proteins were labeled with fluorescein isothiocyanate (FITC, Pierce) according to the manufacturer's instructions. After extensive dialyses against cold PBS was performed to remove free FITC, the concentrations of labeled proteins were adjusted to 0.1 mg/mL. The intracellular presence of FITC-labeled proteins in HEK293T cells was demonstrated by confocal microscopy. The nucleus was stained with DAPI. Scale bars correspond to 50 μm. **(B)** Inhibition of NF-κB activation by CP-TcpAh in HEK293T cells. Activation of the NF-κB-binding promoter in HEK293T cells transfected with NF-κB luciferase reporter (NF-κB-Luc; 250 ng/mL), renilla luciferase reporter (10 ng/mL), and expression vectors for MyD88 (50 ng/mL). After 24 hours, purified CP-TcpAh protein was added into culture medium by indicated concentrations for 12 h. Error bars represent mean ± SD. All data are from at least three independent experiments. $^{**}p < 0.01$, ns, not significant.
